# Enrollment and Retention of Participants in Remote Digital Health Studies: Scoping Review and Framework Proposal

**DOI:** 10.2196/39910

**Published:** 2022-09-09

**Authors:** Paola Daniore, Vasileios Nittas, Viktor von Wyl

**Affiliations:** 1 Institute for Implementation Science in Healthcare University of Zurich Zurich Switzerland; 2 Digital Society Initiative University of Zurich Zurich Switzerland; 3 Epidemiology, Biostatistics and Prevention Institute University of Zurich Zurich Switzerland

**Keywords:** remote digital health studies, remote clinical trials, remote cohorts, digital epidemiology, digital health, health outcome, conceptual framework, user-centered design, population-based digital health, participant recruitment, interventional study

## Abstract

**Background:**

Digital technologies are increasingly used in health research to collect real-world data from wider populations. A new wave of digital health studies relies primarily on digital technologies to conduct research entirely remotely. Remote digital health studies hold promise to significant cost and time advantages over traditional, in-person studies. However, such studies have been reported to typically suffer from participant attrition, the sources for which are still largely understudied.

**Objective:**

To contribute to future remote digital health study planning, we present a conceptual framework and hypotheses for study enrollment and completion. The framework introduces 3 participation criteria that impact remote digital health study outcomes: (1) participant motivation profile and incentives or nudges, (2) participant task complexity, and (3) scientific requirements. The goal of this study is to inform the planning and implementation of remote digital health studies from a person-centered perspective.

**Methods:**

We conducted a scoping review to collect information on participation in remote digital health studies, focusing on methodological aspects that impact participant enrollment and retention. Comprehensive searches were conducted on the PubMed, CINAHL, and Web of Science databases, and additional sources were included in our study from citation searching. We included digital health studies that were fully conducted remotely, included information on at least one of the framework criteria during recruitment, onboarding or retention phases of the studies, and included study enrollment or completion outcomes. Qualitative analyses were performed to synthesize the findings from the included studies.

**Results:**

We report qualitative findings from 37 included studies that reveal high values of achieved median participant enrollment based on target sample size calculations, 128% (IQR 100%-234%), and median study completion, 48% (IQR 35%-76%). Increased median study completion is observed for studies that provided incentives or nudges to extrinsically motivated participants (62%, IQR 43%-78%). Reducing task complexity for participants in the absence of incentives or nudges did not improve median study enrollment (103%, IQR 102%-370%) or completion (43%, IQR 22%-60%) in observational studies, in comparison to interventional studies that provided more incentives or nudges (median study completion rate of 55%, IQR 38%-79%). Furthermore, there were inconsistencies in measures of completion across the assessed remote digital health studies, where only around half of the studies with completion measures (14/27, 52%) were based on participant retention throughout the study period.

**Conclusions:**

Few studies reported on participatory factors and study outcomes in a consistent manner, which may have limited the evidence base for our study. Our assessment may also have suffered from publication bias or unrepresentative study samples due to an observed preference for participants with digital literacy skills in digital health studies. Nevertheless, we find that future remote digital health study planning can benefit from targeting specific participant profiles, providing incentives and nudges, and reducing study complexity to improve study outcomes.

## Introduction

### Background

The widespread availability of smartphones (estimated to be 3.6 billion users worldwide [1]) presents the opportunity to involve diverse population groups in health research. Mobile technologies, such as smartphones and wearables, have come to play a central role in health research, giving rise to digital health studies that are conducted partly or entirely remotely. Although there is no unified definition, we define remote digital health studies as longitudinal studies that use mobile technologies to conduct all key steps of a study completely online [2]. Remote digital health studies promise significant cost, time, and scalability advantages when compared with traditional studies, by allowing key steps of the study investigations to be conducted in real-time and without in-person presence [3,4]. Overcoming the barriers of time and physical presence, remote digital health studies allow for the long-term monitoring of larger populations and thus promise to advance health research and patient care delivery [5-7].

Despite these opportunities, recent studies report high participant attrition rates, likely partially attributable to the lack of in-person interactions between researchers and study participants. Other studies highlight the risk of recruitment bias, especially with younger, more affluent, and often healthier populations being overrepresented in studies with digital technologies [8-10]. These concerns point toward a possible imbalance between participants who typically join remote digital health studies and participants who are often underrepresented, but may benefit the most from remote digital health research and monitoring. This may be a result from a lack of understanding of the motivators, facilitators, and barriers that enable participation in remote digital health studies [11,12].

Trends of participant enrollment and retention have been widely investigated in traditional research settings [13-15] and with digital health studies following Eysenbach’s Law of Attrition [16]. However, there is a paucity of evidence that supports study planning in remote digital health research. Furthermore, most study planning recommendations for remote digital health studies are based on qualitative methods and focus on scientific, rather than participant-specific requirements [17,18]. This presents an unmet need for quantitative, evidence-based guidance that informs remote digital health study planning to enable high enrollment and retention through a person-centered lens.

### Aims

This review aims to explore participant enrollment and retention for remote digital health studies. We introduce a framework on the interplay between 3 criteria explored and validated in previous digital health studies: (1) participant motivation profile and incentives or nudges, (2) participant task complexity, and (3) scientific requirements. We propose hypotheses and explore them with a scoping review of remote digital health studies specifically, focusing on methodological aspects that affect participant enrollment and retention. The goal of this scoping review is to inform the planning and implementation of remote digital health studies from a person-centered perspective.

### Conceptual Framework and Hypotheses

**Figure 1:**
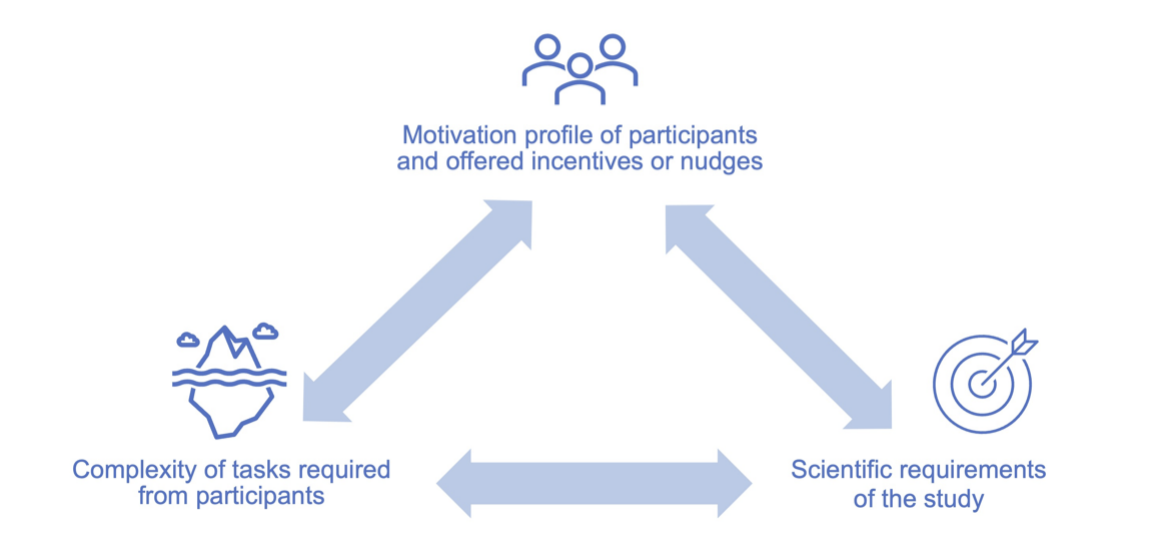
Guiding framework for remote digital health studies.

We introduce a conceptual framework that encompasses the main factors that affect digital health study enrollment and retention from a person-centered lens, to guide the extraction of relevant information. The framework is based on the notion that enrollment and retention in remote digital health studies are influenced by 3 elements: (1) participant motivation profile and incentives or nudges, (2) participant task complexity, and (3) scientific requirements (Figure 1). Our conceptual framework and hypotheses were informed by Eysenbach [16], previous large-scale remote digital health studies [5,19], as well as by our personal experiences in planning and conducting 2 longitudinal remote digital health studies [20,21]. A more detailed description of the framework development process can be found in Multimedia Appendix 1 [2,5,14,16,19-28].

We define hypotheses to explore in this study. Specifically, we expect that incentives and nudges increase participant motivation to enroll in and complete a study (Hypothesis 1). On the contrary, we expect a decrease in enrollment and study completion with increased complexity of study tasks (Hypothesis 2a). Finally, we also expect that participants in interventional studies may be willing to endure higher task complexity than participants enrolled in observational studies (Hypothesis 2b).

### Conceptual Framework Definitions

#### Motivation Profile of Participants and Offered Incentives

Participants’ motivation to enroll and complete remote digital health studies can be *intrinsic* or *extrinsic*. Intrinsic participation may be motivated by altruistic motives or by hopes for medical advances, especially among individuals with chronic diseases. Extrinsic participation can be motivated, among others, by investigators in the form of monetary incentives or clinical referrals [29,30]. The provision of incentives or nudges may help extrinsically motivated participants to enroll and participate in a study. Incentives are predominantly offered in the form of monetary compensation, while nudges mainly come in the form of reminders or personal contact [31-34]. Intrinsically motivated participants do not necessarily require incentives or nudges to enroll or participate in a study. It is to be noted, however, that intrinsic and extrinsic motivations are not necessarily mutually exclusive from one another and may coincide in a study. In this review, we conduct an exploratory assessment based on the aforesaid definitions. Different approaches to assessing participant motivations may exist.

#### Complexity of Tasks Required From Participants

The *frequency* and *complexity* of tasks required from the participants, along with the expected *duration of the study*, can impact study completion. Tasks can be categorized as physical and mental tasks. Typical physical tasks required of participants include physical activity tasks, such as walking a certain number of steps every day, as well as other essential tasks to fulfill the study’s requirements, such as signing an informed consent form, filling out questionnaires at baseline or at follow-up, or logging of health-related outcomes [27,35,36]. Mental tasks, such as the measure of cognitive burden of a participant, are harder to quantify as they typically rely on patient-reported outcome measures [37,38]. In digital heath studies, task complexity is compounded by electronic measurements and device handling, which may require high digital literacy skills [39-41]. Task complexity can be reduced by accompanying measures, such as passive data collection, or technical support. Applied to remote digital health studies, high-complexity tasks come in the form of many required tasks over long study periods. Provision of assistance during participant onboarding may improve digital literacy skills of participants, while also instilling a sense of trust between the participant and the researcher through personal interactions [42,43].

#### Scientific Requirements of the Study

Scientific requirements define the *study design* and expected *target sample size*. Therefore, scientific requirements set the goal of the study, while task complexity or incentives or nudges enable study goal achievement. Target sample sizes are generally estimated through statistical power analyses for enrollment goals [27,44]. Consideration of participant requirements may help increase the statistical power and reduce selection bias of the study. However, sample size calculation methods often may not anticipate participant losses to follow-up and failure to complete tasks [45]. Sensitivity analyses may be conducted after the study to assess the impact of deviations in participant enrollment or retention.

## Methods

### Study Outcome Definitions

In our study we refer to 3 phases of participants’ involvement in remote digital health studies: recruitment, onboarding, and retention (Tables 1 and 2). We summarize the outcomes of the 3 phases of participant involvement in remote digital health studies as study enrollment and completion. A detailed approach for defining each phase of the study and the outcomes can be found in Multimedia Appendix 1.

The 3 criteria in our proposed framework can affect all phases of remote digital health studies. In the next sections, we will explore our hypotheses by assessing the outcomes of interest for each framework criteria across all 3 phases of remote digital health studies.

**Table 1:** Phases of digital health studies.

Phases	Definition
Recruitment	Fulfillment of requirements for study enrollment
Onboarding	Provision of (technical) assistance to start study tasks
Retention	Fulfillment of requirements for study completion

**Table 2:** Outcomes of digital health studies.

Outcomes	Measure
Study enrollment target	(Achieved enrollment/target enrollment) × 100%
Study completion	Percentage of enrolled participants who completed the study

### Search Strategy and Study Selection

To explore our hypotheses, we conducted a scoping review according to the PRISMA-ScR (Preferred Reporting Items for Systematic reviews and Meta-analyses Extension for Scoping Reviews) checklist [46] (Multimedia Appendix 2). Our search was performed on the PubMed, CINAHL, and Web of Science databases for primary research articles published between January 1, 2016, and June 31, 2021. We limited our search to this period based on the results of a preliminary search revealing a paucity of remote digital health studies published before 2016 [19,47]. We also assessed reviews and included relevant primary studies for a full-text review based on citation searches. The complete search strategy for each database can be found in Multimedia Appendix 3.

Our selection was guided by the criteria outlined in Textbox 1. Screening was conducted in 2 phases. Initially, we screened titles and abstracts and then the full texts. For both phases, the entire screening was conducted by one investigator (PD), while a second investigator (VN) performed checks on a randomly selected sample of studies in the title and abstract screening (80/662 articles, 12.1%) and in the full-text screening (50/150, 33.3%). Any disagreements were discussed and, if required, consensus was achieved through the third investigator (VvW). Agreement was 75/80 articles (94%) for title and abstract screening and 45/50 articles (90%) for full-text screening.

**Literature inclusion criteria.**

- Studies that match the definition of a remote digital health study (ie, digital health study where all steps are conducted online and without in-person interactions between participants and study investigators).
- Studies that mention their approach to recruit, onboard, or retain participants.
- Studies that mention approaches to at least one of the proposed framework criteria.
- Studies that provide evidence on study enrollment or study completion.

### Data Extraction and Synthesis

Data extraction was standardized yet developed iteratively. In cases where research articles referenced to the original protocol for the same study, the additional protocols were assessed to collect missing information of interest. The initial data extraction was based on standard study characteristics (eg, study design, participant characteristics) and guided by the conceptual framework (Figure 1). During the full-text screening, other criteria of interest (eg, measure of study completion) were identified as relevant and retrospectively included.

We conducted qualitative analyses to explore our hypotheses. Qualitative analyses are presented for the entire study sample, as well as for samples stratified based on the median study duration. Descriptions of qualitative data were provided to summarize key findings from the included studies within the structure of the conceptual framework. We also conducted an exploratory quantitative assessment of the framework criteria with the study enrollment and completion outcomes. The correlations between measures relevant to our study’s framework criteria and the study enrollment and completion outcomes retrieved from the included studies were assessed using Spearman rank correlation for continuous variables and the Kruskal-Wallis rank-sum test for categorical variables.

All screening and extraction procedures were completed in MS Excel (Microsoft, Inc.). All statistical analyses were completed in R, version 4.0.0 (R Foundation for Statistical Computing) using ggplot2, version 3.3.3, for plots. The threshold for statistical significance was set at *P*<.05 (2-tailed testing).

### Data Availability

The data from the papers that support the findings of this study are publicly available. All data used in this review can be found in Multimedia Appendices 4-6 [6,7,43,48-81].

## Results

### Data Search and Identification of Articles

**Figure 2:**
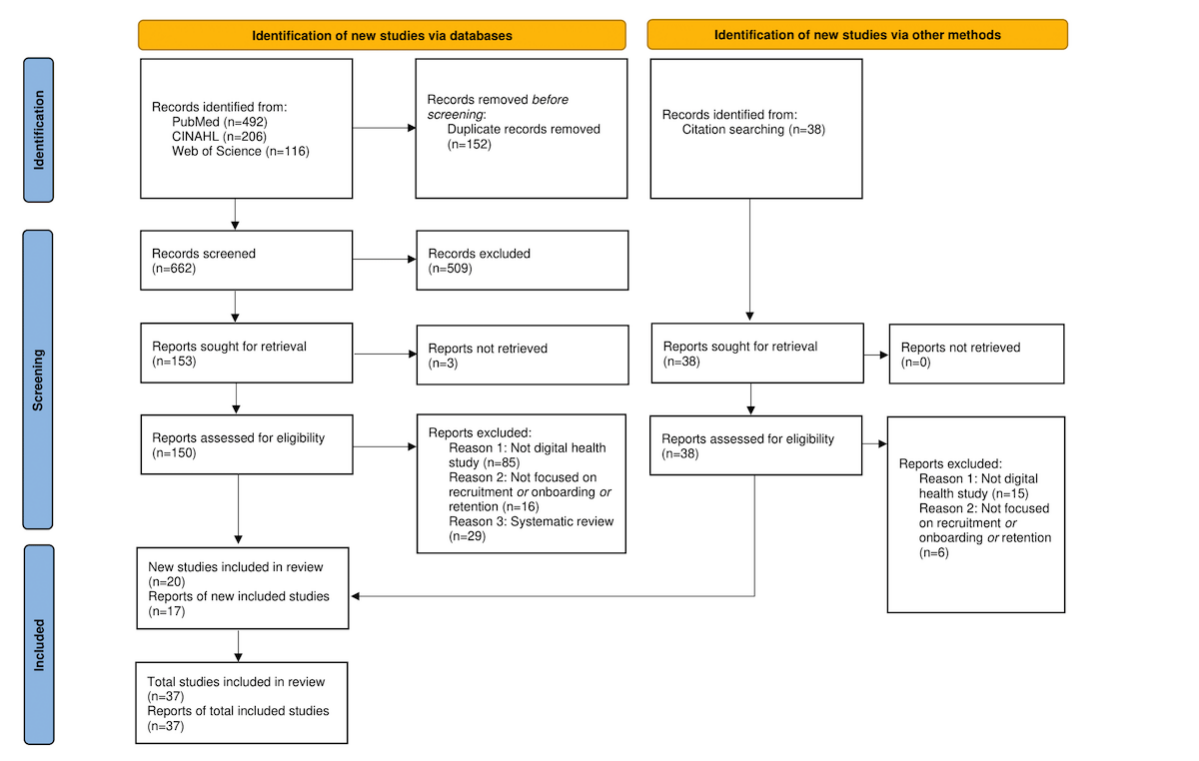
Flowchart for study identification, screening, and inclusion.

Our database searches yielded 662 references (Figure 2). Twenty studies fulfilled all inclusion criteria and were finally included. An additional 38 studies were identified through citation searches in relevant systematic reviews, of which 17 met all inclusion criteria. Overall, 37 studies were included for assessment in this review.

### General Description of Included Studies

The study characteristics, study requirements, and framework criteria outcomes of the final study sample are presented in Multimedia Appendix 1 and summarized in Multimedia Appendices 4-6 [6,7,43,48-81], respectively. Of the 37 included studies, 19/37 (51%) were randomized control trials, 11/37 (30%) nonexperimental studies, and 7/37 (19%) quasi-experimental studies. All studies were longitudinal, 28/37 (76%) of which were interventional and 9/37 (24%) observational studies.

The most prevalent therapeutic areas in our study sample were psychiatry (11/37 studies, 30%), neurology (4/37 studies, 11%), and addiction (4/37 studies, 11%). Most studies (12/37, 32%) measured physical activity levels, 3/37 (8%) measured smoking cessation, 3/37 (8%) measured depression management, 2/37 (5%) measured stress management, 2/37 (5%) measured pain management, and the rest of the studies (15/37, 41%) measured other outcomes. Most studies relied on smartphones for the study procedure (23/37, 62%) and predominantly measured step counts (7/37, 19%). Most studies (34/37, 92%) collected patient-reported outcomes.

Most studies (25/37, 68%) targeted participants with an existing health condition, mainly enrolled females (median female enrollment of 77%, IQR 52%-88%) and participants with a median age of 39 years (IQR 35-47 years). A median of 43% (IQR 32-49%) of enrolled participants had a lower educational background, while information on enrolled participant income (27/37, 73%) and employment status (25/37, 68%) was largely missing from the studies.

### Description of Included Studies According to Framework

A description of the included studies based on our framework’s criteria is found in Table 3. The table summarizes the participants’ motivation profiles, the most prevalent incentives and nudges provided at each stage of the study (more information is available in Multimedia Appendix 6), the frequencies of the tasks for the studies and measures provided to reduce participant burden, the target sample size of the studies, and the study outcomes. Given the scarcity of mental task measures in the assessed studies, our review only included measures of physical tasks. The table is stratified by the median duration of the included studies (12 weeks, IQR 12-26 weeks).

A total of 27 studies reported on study completion (Multimedia Appendix 5); 14/27 (52%) studies assessed for completion based on participant retention throughout the study period, 10/27 (37%) studies assessed for completion based on researcher-defined metrics (eg, completion of 1 task within a 30-day period) and 3/27 (11%) studies assessed for completion based on the fulfillment of all study tasks.

In the next sections, study enrollment and completion are assessed based on our study’s hypotheses. An assessment of the task frequencies and study durations is made against measures provided to reduce participant burden, as well as incentives or nudges provided to participants. Further descriptions and correlations between the studies’ framework criteria and outcomes are reported in Multimedia Appendix 7.

**Table 3:** Summary statistics of included studies based on framework criteria.

Profile				Study duration^a^: ≤12 weeks (n=19)		Study duration: >12 weeks (n=15)		Study duration: unknown duration (n=3)			Overall (n=37)		
**Motivation profile of participants and offered incentives or nudges**													
	**Motivation profiles, n (%)**												
		Extrinsic	14 (74)		8 (53)		0 (0)		22 (59)				
		Intrinsic	5 (26)		7 (47)		3 (100)		15 (41)				
	**Incentives/nudges: recruitment^b^, n (%)**												
		Monetary	4 (21)		3 (20)		2 (67)		9 (24)				
		Referral source	3 (16)		1 (7)		0 (0)		4 (11)				
		Vested interest	0 (0)		2 (13)		1 (33)		3 (8)				
	**Incentives/nudges: onboarding, n (%)**												
		Personal assistance	1 (5)		5 (33)		0 (0)		6 (16)				
		Peer support	0 (0)		1 (7)		0 (0)		1 (3)				
	**Incentives/nudges: retention, n (%)**												
		Monetary	10 (53)		6 (40)		1 (33)		15 (41)				
		Reminders	10 (53)		5 (33)		0 (0)		15 (41)				
		Personal contact	9 (47)		3 (20)		0 (0)		12 (32)				
	Incentives/nudges: minimum one phase^c^, n (%)		18 (95)		13 (87)		2 (67)			33 (89)			
**Complexity of tasks required from participants**													
	**Task complexity: study tasks**												
		Monthly steps, median (IQR)^d^	16 (5-30)		30 (29-36)		—^e^		28 (8-31)				
		Total steps, median (IQR)^f^	35 (12-93)		99 (14-180)		3.00 (3.0-3.0)		58 (10-120)				
	**Task complexity: reduction of burden, n (%)**												
		Passive monitoring	7 (37)		9 (60)		0 (0)		16 (43)				
		Short, repetitive daily tasks	6 (32)		8 (53)		0 (0)		14 (38)				
		At least one burden reduction^g^	9 (47)		12 (80)		0 (0)		21 (57)				
**Scientific requirements of the study**													
	Target sample size, median (IQR)		72 (50-120)		313 (238-838)		473 (336-609)			200 (50-350)			
**Study outcomes**													
	Enrolled participants, median (IQR)		281 (89-450)		560 (150-2800)		100 (55-200)			300 (89-950)			
	Retained participants, median (IQR)		110 (45-240)		800 (190-1700)		—			180 (70-690)			
	Enrollment target (%), median (IQR)		150 (124-302)		101 (96-125)		82 (48-116)			128 (100-234)			
	Study completion (%), median (IQR)		48 (38-73)		55 (32-79)		—			48 (35-76)			

^a^The duration of the study as defined in the study protocol.

^b^The top 3 recruitment and retention incentives and nudges are reported; more information is available in Multimedia Appendix 6.

^c^Incentive or nudge provided in at least one of the study phases: recruitment, onboarding, or retention.

^d^The number of physical tasks investigators required participants to do on a monthly basis throughout the study duration as defined in the study protocol.

^e^Not available.

^f^The total number of physical tasks investigators required participants to do throughout the study duration as defined in the study protocol.

^g^Burden reduction for participants either through 1 of the 2 approaches, passive monitoring or short, repetitive daily tasks, provided in a study.

### Evidence for Hypotheses

#### Hypothesis 1: Study Outcomes Based on Motivation Profiles and Incentives or Nudges

**Figure 3:**
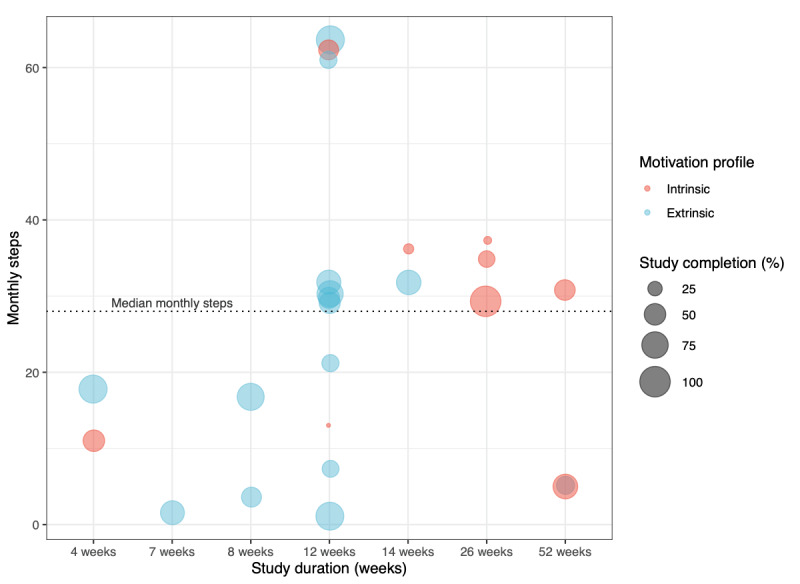
Study completion based on participant motivations and study requirements.

In this section we link evidence from the studies’ *task complexity* with the *participant motivation profile and incentives or nudges* criteria of our framework to assess study enrollment and completion outcomes. The 15 studies that enrolled intrinsically motivated participants reached a median enrollment target of 137% (IQR 98%-226%), and the 22 studies that enrolled extrinsically motivated participants reached a median enrollment target of 126% (IQR 102%-213%). The median completion rate of studies that focused on intrinsically motivated participants was 41% (IQR 20%-49%), whereas those that included extrinsically motivated participants had a median study completion of 62% (IQR 43%-78%; Figure 3).

Studies that enrolled intrinsically motivated participants had a median duration of 14 weeks (IQR 12-26) and had participants complete a median of 30 (IQR 12-36) tasks per month. Approximately half of these studies (7/15, 47%) offered passive monitoring of health data or had participants complete lower complexity, repetitive daily tasks (7/15, 47%).

Studies that enrolled extrinsically motivated participants had a median duration of 12 weeks (IQR 12-14) and had participants complete a median of 26 (IQR 7-30) tasks per month. Some of the studies (9/22, 41%) offered passive monitoring of health data and fewer offered participants lower complexity, repetitive daily tasks (7/22, 32%).

Incentives or nudges for recruitment of intrinsically motivated participants were offered only in 4/15 (27%) studies and incentives or nudges for retention were offered in 9/15 (60%) studies. For studies that targeted extrinsically motivated participants, incentives or nudges for recruitment were offered in 12/22 (55%) studies and incentives or nudges for retention were offered in all (22/22, 100%) studies.

Statistical significance testing did not reveal evidence for an effect of intrinsic or extrinsic study motivations or the provision of incentives and nudges on study enrollment or completion outcomes (Multimedia Appendix 7).

#### Hypothesis 2: Study Outcomes Based on Task Complexity and Study Design

In this section we link evidence from the study *task complexity* with the *scientific requirements* criteria of our framework to assess study enrollment and completion outcomes.

**Figure 4:**
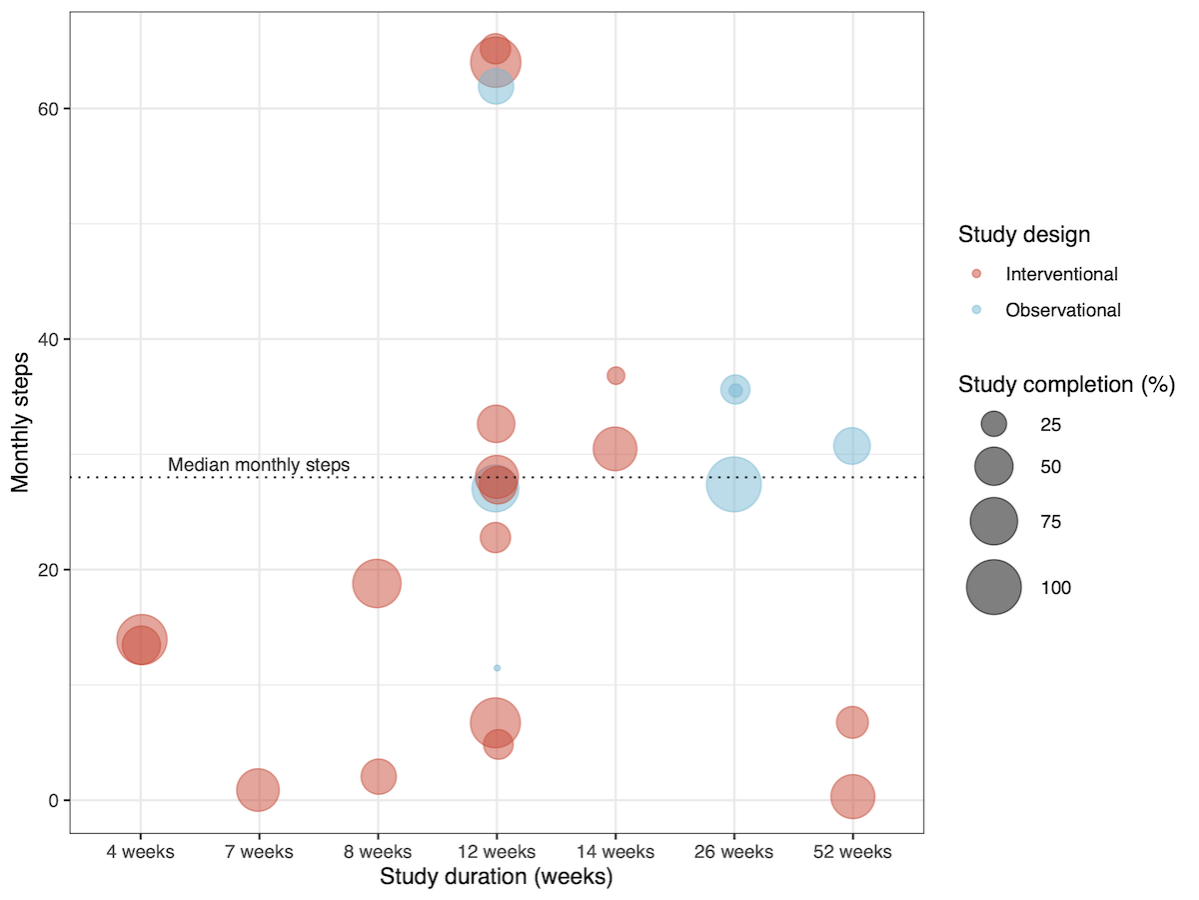
Study completion based on study design and requirements.

Interventional studies achieved a median enrollment target of 128% (IQR 100%-150%) and observational studies a median enrollment target of 103% (IQR 102%-370%). Interventional studies achieved a median study completion of 55% (IQR 38%-79%) and observational studies achieved a median study completion of 43% (IQR 22%-60%; Figure 4).

A total of 28 interventional studies had a median duration of 12 weeks (IQR 12-14) and had participants complete a median of 16 (IQR 4-30) tasks per month. Few interventional studies (8/28, 29%) offered passive monitoring of health data and had participants complete lower complexity, repetitive daily tasks (8/28, 29%).

Nine observational studies had a median duration of 26 weeks (IQR 12-26 weeks) and required participants to complete a median of 30 (IQR 29-35) tasks per month. Most observational studies (7/9, 78%) offered passive monitoring of health data and had participants complete lower complexity, repetitive daily tasks (6/9, 67%).

Approximately half of the interventional studies (15/28, 54%) provided incentives or nudges for recruitment, and 26 interventional studies (26/28, 93%) provided incentives or nudges for retention. Only 1 observational study (1/9, 11%) provided an incentive for recruitment, while 5 (5/9, 56%) of the observational studies provided incentives or nudges for participant retention.

Statistical analyses did not reveal evidence for an effect of task complexity or study design on study enrollment or completion outcomes (Multimedia Appendix 7). The observed coefficients only suggest weak or no correlations, which were not statistically significant (Multimedia Appendix 7).

## Discussion

### Principal Findings

The studies from our sample were primarily interventional. Most studies targeted extrinsically motivated participants for shorter-length studies and provided incentives or nudges to recruit or retain participants. Around half of the studies provided measures to reduce participant burden through passive data collection or by requiring participants to complete frequent, shorter tasks. The study sample revealed high values of achieved target participant enrollment and retention. These findings suggest that the criteria defined in our framework may have an impact on the studies’ enrollment and completion outcomes.

Exploring our first hypothesis, high study enrollment is observed in studies that targeted intrinsically motivated participants. Study completion in these studies was lower than in those that targeted extrinsically motivated participants. This may be due to researchers requiring intrinsically motivated participants to complete more tasks per month in comparison to studies with extrinsically motivated participants or due the provision of additional incentives in studies with extrinsically motivated participants. Furthermore, the provision of lower complexity, daily repetitive tasks was similar between studies with intrinsically or extrinsically motivated participants. Our study’s preliminary qualitative analyses, although the results did not reach statistical significance, indicate that the provision of incentives or nudges as well as the reduction of required tasks from participants may contribute to higher study completion.

Exploring our second hypothesis, differences in study enrollment and completion outcomes are also observed between interventional and observational study designs. Here, higher enrollment is observed in interventional studies, despite higher efforts from most of the observational studies in our sample to achieve wider reach study recruitment and simplify enrollment procedures. Interventional studies revealed a higher study completion, although fewer measures to reduce participant burden were provided. We find that our framework provides preliminary relevant criteria and findings relevant to remote digital health study participation to guide researchers in study planning.

### Comparison With Prior Work

#### Descriptive Assessment of Participation Strategies in Remote Digital Health Studies

Descriptive results from our study sample reveal a preference for investigators to provide incentives and nudges to retain rather than recruit participants. Researchers’ choice to focus on retaining participants could be in response to reported high attrition rates in digital health studies [15,83-85]. If participants are not retained, the quality of the evidence base of a study is decreased. From our study sample, the lower provision of incentives or nudges at recruitment could have likely been motivated by higher participant reach through open social networks, multimodal recruitment strategies, and user-friendly interfaces [48-64,80,81]. The replacement of in-person enrollment procedures with mobile-forward procedures that are easy to use, specifically, enables easier study enrollment, as mentioned in 8 of our studies [6,50,53,55-57,65,66]. However, it is important to consider that successful recruitment strategies do not necessarily translate into high participant retention. This could be due, in part, to unrepresentative samples that enroll in a study to collect financial incentives and then dropout [86,87]. Studies may be highly effective if they place equal importance on their recruitment and retention strategies, while applying sample validation approaches to ensure the representativeness of their study sample.

Studies of shorter durations reported lower study completion than those of longer durations. This is inconsistent with the observation that studies that ran for 12 weeks or less required less tasks to be completed by participants than in studies’ that ran for over 12 weeks. However, half of the longer duration studies reduced participant burden by asking them to complete repetitive, short-length tasks once a day [52,54,56,62,67-69,80]. For most of these studies, these tasks could be completed at any point in time during the day and the completion of all tasks was made optional. The majority of longer duration studies also collected data passively through smartphones or wearable sensors [7,48,51,54,60,61,67,70,71]. These findings are in line with recent literature on best practices to maximize participation in longitudinal digital health studies [88,89]. Specifically, evidence aligns with our observations that the integration of short, repetitive tasks that align with participants’ daily lives is more likely to achieve higher participation in comparison to longer, infrequent tasks with higher cognitive burden [49,72,90].

Few studies in our sample involved onboarding procedures for participants. This may be due to investigators’ possible preference for in-person interactions during study enrollment. Previous literature on this topic suggests that interacting with participants virtually, rather than in person, may contribute to higher study attrition [16,83,91]. However, a growing body of recent literature points to the potential of leveraging a combination of user-centered methodologies and easier user interfaces to personalize study designs and maintain high engagement through personal (remote) contact by allowing participants to build trust with researchers [92-95]. Furthermore, survey responses from 2 studies in our cohort that compared in-person and remote procedures reported high participant satisfaction with the remote interactions with investigators [43,51]. Nevertheless, a more thorough investigation on the role of in-person versus remote onboarding procedures is missing in the literature. This calls attention to a potentially essential step in remote digital health research that is understudied yet could significantly impact study participation.

#### Exploration of Hypotheses From Relationships Between Framework Criteria and Outcomes

On average, the studies in our sample enrolled more participants than their target sample size calculations, with studies that ran for 12 weeks or less achieving higher enrollment targets than studies that ran for longer than 12 weeks. The median study completion rate was approximately 50%, which is relatively high when considering that retention rates as low as 10% are common in digital health research [7,28,96].

A description of our study sample provides indications of a possible higher study completion for studies that provide incentives or nudges. This was observed with interventional studies that, despite providing fewer lower complexity tasks from participants than the observational studies in our sample, managed to achieve higher study completion. The higher study completion could be due to interventional studies providing more incentives or nudges to participants than observational studies. The provision of incentives in studies is consistent with recent approaches that use willingness to accept estimations or incentive matching with local wages to enable higher study enrollment and completion [97-101]. Furthermore, the choice of offering nudges to study participants is supported by recent adaptations to the nudge theory, which claims that providing motivational elements in digital health research may affect decision making in study participants [102]. In recent studies, different forms of nudges, or motivational strategies, such as gamification, personalization of the digital solution, and peer support (eg, through citizen science methodologies [2]), have been reported as successful in maintaining high user retention [103-105]. The creation of online communities and support groups was also mentioned in 9 of our studies as a key contributor for participant retention [49,51,60,62,65,70,73,74,81].

Enrollment for interventional studies was higher than for observational studies. This was observed despite wider reach study recruitment and easier enrollment procedures efforts through digital recruitment channels and mobile technologies observed in most observational studies from our sample [49,54,61,75,76]. Observational studies also achieved lower completion outcomes despite providing tasks that reduce participant burden, which points to a possible lack of appropriate nudges to avoid participants dropping out after enrollment. It was also observed that studies focused on intrinsically motivated participants enrolled participants for longer studies and required them to complete more tasks, while providing less incentives or nudges than in studies focused on extrinsically motivated participants. This may have contributed to selection of specific participant profiles and higher loss to follow up, as evidenced by lower completion of studies with intrinsically motivated participants. Conversely, the studies with intrinsically motivated participants that achieved study completion values higher than the median of this group of studies predominantly focused on participants at risk or with chronic conditions in an observational study format [54,67,69,71,75]. This observation aligns with recent literature on the prominent role of disease status in enabling higher study retention in digital health studies through intrinsic motivations [19,106,107].

Our analyses yielded no statistical evidence to support our hypothesis of an interaction between factors that affect participation in remote digital health studies and the study outcomes. However, the lack of statistical support for the other criteria does not invalidate our hypotheses. First, only few remote digital health studies were identified that provided detailed information on approaches used to enhance study enrollment and retention in their methodologies. This warrants more systematic reporting of remote digital health study operations. Second, publication bias may have influenced our statistical calculations. For example, studies that failed to reach the target sample size may not have gotten published in peer-reviewed journals. This may have biased our correlations toward the null hypothesis. Third, there were observed preferences for the target participant profiles (eg, levels of digital literacy) and the study procedure that likely had an impact on the studies’ outcomes. To enable future assessments that evaluate whether specific participation factors, such as mental tasks that impact cognitive burden, have an impact on study outcomes, it is recommended to report these factors individually in remote digital health studies. The availability of these data could enable more comprehensive reviews that can thoroughly investigate these interactions through statistical analyses, as well as more in-depth explorations of participation enablers and inhibitors through digital survey studies.

#### Inconsistencies of Study Completion Measures

Heterogeneity in researchers’ choices to measure study completion was observed in our sample. Although all the studies we assessed outlined the expected tasks for the study participants to complete in their methodologies (or in referenced larger studies or protocols [55,108-115]), only 3 measured study completion based on the achievement of all tasks defined in the study [64,77,81]. Interestingly, 9 studies measured study completion based on researcher-defined criteria for task completion (eg, completion of 1 task within a 30-day period) specific to their study design [49,53-55,60,61,73,75,80]. Given the novelty of digital health research, the choice of different study completion metrics by researchers is not surprising. However, this poses difficulties in allowing for direct comparisons between studies, as the former approach can be regarded as more conservative, while the latter more lenient. It is, therefore, important that study completion metrics are studied in more detail to determine whether a one-size-fits-all approach should be taken for all studies, or if bespoke approaches to measure completion are more suitable.

### Future Directions for Remote Digital Health Study Planning

Although our statistical analyses were inconclusive, we conducted an initial exploratory assessment of the study’s qualitative data. We adapted the conceptual framework based on best practices found from an assessment of our study sample’s descriptive results that can inform future planning of remote digital health studies:

- Adapt incentives and nudges provided to participants based on their motivation profile: offer different incentives or nudges at each key step of the study procedure. Monetary incentives may contribute to higher study enrollment [48,53,55-57,70,78], after sample validation [86,87], whereas nudges in the form of assistance during onboarding [48,55] and the provision of reminders [50,53,55,60,64,73] or a participant community (eg, through citizen science [2]) could contribute to higher retention. As technology replaces in-person interactions, the procedures set in place should be user-friendly [50,53,55-57,65,66] and enable participants to build personal relationships, with either study participants or study investigators [116]. An adequate assessment of participant profiles and their motivations to join the study can help adapt the provision of incentives or nudges. It is recommended that study investigators do not underestimate the requirement for additional incentives or nudges with intrinsically motivated participants in their study planning.
- Reduce and simplify the study’s tasks: reducing the number of tasks required from participants in combination with providing incentives may achieve higher enrollment and completion in studies with intrinsically motivated participants, especially if they align with participants’ daily lives [53-55,60,78,79]. The use of digital tools with simpler user interfaces and passive monitoring can also facilitate task completion by reducing participants’ cognitive burden [49,54,60,73,78,79]. The implementation of onboarding procedures may also increase participants’ trust, understanding of, and confidence in the study [43,51]. Based on our preliminary assessment of the upper quantile of our results, the required number of physical tasks in studies with intrinsically motivated participants should ideally not exceed 36 steps per month.
- Broaden the scientific requirements: adjust the design of the investigation and target sample size by simplifying the study’s research question. Broader research questions that affect larger population groups may help with achieving a representative study sample, which can be generalized to wider populations [49,54,61,67,75]. Study designs that consider participant motivations, the provision of incentives or nudges, and task complexities can contribute to higher study enrollment and completion outcomes.

We also suggest more systematic reporting on these criteria for study enrollment and completion to facilitate further quantitative assessments and knowledge exchange. This is particularly relevant because, compared with traditional health studies, remote digital health studies bring upon additional, less well-studied challenges. To facilitate the development of remote digital health study methodologies, revised study planning approaches, as voiced previously by others [16], are necessary. This is also of relevance for hybrid or fully decentralized trials, where their reliance on remote procedures also affects participation [117,118]. There is also a need for systematic reporting of additional procedural aspects of study execution with remote digital health studies. Specifically, different reporting requirements are encouraged, such as adapted sample size calculations, approaches to remote recruitment, and onboarding of participants in the “Methods” sections of studies, as well as detailed participant descriptions and aligned reporting of study completion measures in the “Results” sections of studies. The formulation of reporting guidelines, similar to STROBE (STrengthening the Reporting of OBservational studies in Epidemiology) [119], mERA (mobile health [mHealth] evidence reporting and assessment) guidelines [120], and CONSORT-EHEALTH standards [27], as well as further refining our conceptual framework with more evidence as it is made available, could be a first step in this direction.

### Strengths and Limitations

To our knowledge, our study is the first in the field that provides a comprehensive overview of the facilitators and barriers to participation in remote digital health studies. Our study is also the first to provide evidence-based guidelines to inform future remote digital health study planning. However, our study has limitations. First, there is no unified definition of a remote digital health study. As such, a broader definition could have yielded more studies of interest from our search strategy. Second, the analysis is reliant on inconsistent criteria for the outcomes of interest defined by the investigators of our study sample, such as approaches to calculate study completion. In this study, we grouped these criteria into 3 categories of outcome measures, however, a comparison of study outcomes with full accuracy was not possible. Third, the studies are classified based on criteria and assumptions defined by the investigators (eg, definition of task complexity), which we attempted to keep as broad as possible based on available literature in the field. Nevertheless, other variables as well as measures for the classifications and definitions could be possible. Fourth, the analyses were conducted based on a limited set of hypotheses defined in this study; more hypotheses could also be possible. Lastly, our study’s dual review approach, based on random screenings, instead of a complete dual screening, may have led to missing studies in our final study sample.

### Conclusion

In this study, we describe a conceptual framework to introduce criteria that affect remote digital health study participation from a person-centered lens. We apply this framework to remote digital health studies to explore hypotheses on the factors that affect participation outcomes. The compiled data from our scoping review reveal that targeting specific participant profiles, the provision of incentives and nudges, or the reduction of study complexity at any stage of the study may improve study outcomes. Future remote digital health study planning requires a focus on participant requirements, as well as broadening scientific requirements to increase participation in studies. Our proposed framework provides an initial structure to facilitate remote digital health study planning, but we highlight the need for systematic reporting guidelines to enable further assessments and knowledge exchange.

## Appendix

Multimedia Appendix 1 Methods and additional descriptive results.

Multimedia Appendix 2 PRISMA-ScR checklist. PRISMA-ScR: Preferred Reporting Items for Systematic reviews and Meta-analyses Extension for Scoping Reviews.

Multimedia Appendix 3 Search strategy. Also see Multimedia Appendix 2.

Multimedia Appendix 4 General characteristics of included studies.

Multimedia Appendix 5 Requirements and outcomes of included studies.

Multimedia Appendix 6 Incentives or nudges and task complexity of included studies.

Multimedia Appendix 7 Associations between framework criteria and outcomes.

## Abbreviations

CONSORT-EHEALTH: Consolidated Standards of Reporting Trials of Electronic and Mobile HEalth Applications and onLine TeleHealth
mERA: mobile health (mHealth) evidence reporting and assessment
PRISMA-ScR: Preferred Reporting Items for Systematic reviews and Meta-analyses Extension for Scoping Reviews
STROBE: STrengthening the Reporting of OBservational studies in Epidemiology

## References

[1] Global Smartphones Sales May Have Peaked Internet. International Monetary Fund.

[2] Puhan MA, Steinemann N, Kamm CP, Müller S, Kuhle J, Kurmann R, Calabrese P, Kesselring J, von Wyl V, Swiss Multiple Sclerosis Registry Smsr (2018). A digitally facilitated citizen-science driven approach accelerates participant recruitment and increases study population diversity. Swiss Med Wkly.

[3] Anguera JA, Jordan JT, Castaneda D, Gazzaley A, Areán PA (2016). Conducting a fully mobile and randomised clinical trial for depression: access, engagement and expense. BMJ Innov.

[4] Inan OT, Tenaerts P, Prindiville SA, Reynolds HR, Dizon DS, Cooper-Arnold K, Turakhia M, Pletcher MJ, Preston KL, Krumholz HM, Marlin BM, Mandl KD, Klasnja P, Spring B, Iturriaga E, Campo R, Desvigne-Nickens P, Rosenberg Y, Steinhubl SR, Califf RM (2020). Digitizing clinical trials. NPJ Digit Med.

[5] Perez MV, Mahaffey KW, Hedlin H, Rumsfeld JS, Garcia A, Ferris T, Balasubramanian V, Russo AM, Rajmane A, Cheung L, Hung G, Lee J, Kowey P, Talati N, Nag D, Gummidipundi SE, Beatty A, Hills MT, Desai S, Granger CB, Desai M, Turakhia MP, Apple Heart Study Investigators (2019). Large-Scale Assessment of a Smartwatch to Identify Atrial Fibrillation. N Engl J Med.

[6] Bot BM, Suver C, Neto EC, Kellen M, Klein A, Bare C, Doerr M, Pratap A, Wilbanks J, Dorsey ER, Friend SH, Trister AD (2016). The mPower study, Parkinson disease mobile data collected using ResearchKit. Sci Data.

[7] Crouthamel M, Quattrocchi E, Watts S, Wang S, Berry P, Garcia-Gancedo L, Hamy V, Williams RE (2018). Using a ResearchKit Smartphone App to Collect Rheumatoid Arthritis Symptoms From Real-World Participants: Feasibility Study. JMIR Mhealth Uhealth.

[8] Nouri SS, Adler-Milstein J, Thao C, Acharya P, Barr-Walker J, Sarkar U, Lyles C (2020). Patient characteristics associated with objective measures of digital health tool use in the United States: A literature review. J Am Med Inform Assoc.

[9] Langford A, Orellana K, Kalinowski J, Aird C, Buderer N (2020). Use of Tablets and Smartphones to Support Medical Decision Making in US Adults: Cross-Sectional Study. JMIR Mhealth Uhealth.

[10] Langford AT, Solid CA, Scott E, Lad M, Maayan E, Williams SK, Seixas AA (2019). Mobile Phone Ownership, Health Apps, and Tablet Use in US Adults With a Self-Reported History of Hypertension: Cross-Sectional Study. JMIR Mhealth Uhealth.

[11] Sezgin E, Özkan-Yildirim S, Yildirim S (2017). Investigation of physicians' awareness and use of mHealth apps: A mixed method study. Health Policy and Technology.

[12] Lennon MR, Bouamrane M, Devlin AM, O'Connor S, O'Donnell C, Chetty U, Agbakoba R, Bikker A, Grieve E, Finch T, Watson N, Wyke S, Mair FS (2017). Readiness for Delivering Digital Health at Scale: Lessons From a Longitudinal Qualitative Evaluation of a National Digital Health Innovation Program in the United Kingdom. J Med Internet Res.

[13] Neve MJ, Collins CE, Morgan PJ (2010). Dropout, nonusage attrition, and pretreatment predictors of nonusage attrition in a commercial Web-based weight loss program. J Med Internet Res.

[14] Pedersen DH, Mansourvar M, Sortsø C, Schmidt T (2019). Predicting Dropouts From an Electronic Health Platform for Lifestyle Interventions: Analysis of Methods and Predictors. J Med Internet Res.

[15] Van der Mispel C, Poppe L, Crombez G, Verloigne M, De Bourdeaudhuij I (2017). A Self-Regulation-Based eHealth Intervention to Promote a Healthy Lifestyle: Investigating User and Website Characteristics Related to Attrition. J Med Internet Res.

[16] Eysenbach G (2005). The law of attrition. J Med Internet Res.

[17] (2021). Global strategy on digital health 2020-2025. World Health Organization.

[18] Chu L, Shah A, Rouholiman D, Riggare S, Gamble J (2018). Patient-Centric Strategies in Digital Health. Digital Health. Health Informatics.

[19] Pratap A, Neto EC, Snyder P, Stepnowsky C, Elhadad N, Grant D, Mohebbi MH, Mooney S, Suver C, Wilbanks J, Mangravite L, Heagerty PJ, Areán P, Omberg L (2020). Indicators of retention in remote digital health studies: a cross-study evaluation of 100,000 participants. NPJ Digit Med.

[20] Corona Immunitas.

[21] Steinemann N, Kuhle J, Calabrese P, Kesselring J, Disanto G, Merkler D, Pot C, Ajdacic-Gross V, Rodgers S, Puhan MA, von Wyl V, Swiss Multiple Sclerosis Registry (2018). The Swiss Multiple Sclerosis Registry (SMSR): study protocol of a participatory, nationwide registry to promote epidemiological and patient-centered MS research. BMC Neurol.

[22] Berwick Donald M (2003). Disseminating innovations in health care. JAMA.

[23] Robinson Ja, Kocman D, Speyer O, Gerasopoulos E (2021). Meeting volunteer expectations — a review of volunteer motivations in citizen science and best practices for their retention through implementation of functional features in CS tools. Journal of Environmental Planning and Management.

[24] Sieverink Floor, Kelders Saskia M, van Gemert-Pijnen Julia Ewc (2017). Clarifying the Concept of Adherence to eHealth Technology: Systematic Review on When Usage Becomes Adherence. J Med Internet Res.

[25] Christensen Helen, Griffiths Kathleen M, Farrer Louise (2009). Adherence in internet interventions for anxiety and depression. J Med Internet Res.

[26] Heinsch Milena, Wyllie Jessica, Carlson Jamie, Wells Hannah, Tickner Campbell, Kay-Lambkin Frances (2021). Theories Informing eHealth Implementation: Systematic Review and Typology Classification. J Med Internet Res.

[27] Eysenbach G, CONSORT-EHEALTH Group (2011). CONSORT-EHEALTH: improving and standardizing evaluation reports of Web-based and mobile health interventions. J Med Internet Res.

[28] van Mierlo Trevor (2014). The 1% rule in four digital health social networks: an observational study. J Med Internet Res.

[29] Milward J, Drummond C, Fincham-Campbell S, Deluca P (2018). What makes online substance-use interventions engaging? A systematic review and narrative synthesis. Digit Health.

[30] de Ridder M, Kim J, Jing Y, Khadra M, Nanan R (2017). A systematic review on incentive-driven mobile health technology: As used in diabetes management. J Telemed Telecare.

[31] Thaler R.H., Sunstein C.R. (2008). Nudge: Improving decisions about health, wealth, and happiness. Const Polit Econ.

[32] Dai H, Saccardo S, Han MA, Roh L, Raja N, Vangala S, Modi H, Pandya S, Sloyan M, Croymans DM (2021). Behavioural nudges increase COVID-19 vaccinations. Nature.

[33] Horne BD, Muhlestein JB, Lappé DL, May HT, Le VT, Bair TL, Babcock D, Bride D, Knowlton KU, Anderson JL (2022). Behavioral Nudges as Patient Decision Support for Medication Adherence: The ENCOURAGE Randomized Controlled Trial. Am Heart J.

[34] Bauer B, Tucker R, Capron D (2019). A Nudge in a New Direction: Integrating Behavioral Economic Strategies Into Suicide Prevention Work. Clinical Psychological Science.

[35] Nittas V, Puhan MA, von Wyl V (2021). Toward a Working Definition of eCohort Studies in Health Research: Narrative Literature Review. JMIR Public Health Surveill.

[36] Nebeker C, Gholami M, Kareem D, Kim E (2021). Applying a Digital Health Checklist and Readability Tools to Improve Informed Consent for Digital Health Research. Front Digit Health.

[37] Torous J, Michalak EE, O'Brien HL (2020). Digital Health and Engagement-Looking Behind the Measures and Methods. JAMA Netw Open.

[38] Taylor KI, Staunton H, Lipsmeier F, Nobbs D, Lindemann M (2020). Outcome measures based on digital health technology sensor data: data- and patient-centric approaches. NPJ Digit Med.

[39] Mackert M, Mabry-Flynn A, Champlin S, Donovan EE, Pounders K (2016). Health Literacy and Health Information Technology Adoption: The Potential for a New Digital Divide. J Med Internet Res.

[40] El Benny M, Kabakian-Khasholian T, El-Jardali F, Bardus M (2021). Application of the eHealth Literacy Model in Digital Health Interventions: Scoping Review. J Med Internet Res.

[41] Rowsell A, Muller I, Murray E, Little P, Byrne CD, Ganahl K, Müller G, Gibney S, Lyles CR, Lucas A, Nutbeam D, Yardley L (2015). Views of People With High and Low Levels of Health Literacy About a Digital Intervention to Promote Physical Activity for Diabetes: A Qualitative Study in Five Countries. J Med Internet Res.

[42] Figueroa CA, Murayama H, Amorim PC, White A, Quiterio A, Luo T, Aguilera A, Smith ADR, Lyles CR, Robinson V, von Vacano C (2022). Applying the Digital Health Social Justice Guide. Front Digit Health.

[43] Hernandez-Ramos R, Aguilera A, Garcia F, Miramontes-Gomez J, Pathak LE, Figueroa CA, Lyles CR (2021). Conducting Internet-Based Visits for Onboarding Populations With Limited Digital Literacy to an mHealth Intervention: Development of a Patient-Centered Approach. JMIR Form Res.

[44] Barnett I, Torous J, Reeder HT, Baker J, Onnela J (2020). Determining sample size and length of follow-up for smartphone-based digital phenotyping studies. J Am Med Inform Assoc.

[45] Whitley E, Ball J (2002). Statistics review 4: sample size calculations. Crit Care.

[46] Tricco AC, Lillie E, Zarin W, O'Brien KK, Colquhoun H, Levac D, Moher D, Peters MD, Horsley T, Weeks L, Hempel S, Akl EA, Chang C, McGowan J, Stewart L, Hartling L, Aldcroft A, Wilson MG, Garritty C, Lewin S, Godfrey CM, Macdonald MT, Langlois EV, Soares-Weiser K, Moriarty J, Clifford T, Tunçalp Ö, Straus SE (2018). PRISMA Extension for Scoping Reviews (PRISMA-ScR): Checklist and Explanation. Ann Intern Med.

[47] Loncar-Turukalo T, Zdravevski E, Machado da Silva J, Chouvarda I, Trajkovik V (2019). Literature on Wearable Technology for Connected Health: Scoping Review of Research Trends, Advances, and Barriers. J Med Internet Res.

[48] Damschroder LJ, Buis LR, McCant FA, Kim HM, Evans R, Oddone EZ, Bastian LA, Hooks G, Kadri R, White-Clark C, Richardson CR, Gierisch JM (2020). Effect of Adding Telephone-Based Brief Coaching to an mHealth App (Stay Strong) for Promoting Physical Activity Among Veterans: Randomized Controlled Trial. J Med Internet Res.

[49] Bailey JF, Agarwal V, Zheng P, Smuck M, Fredericson M, Kennedy DJ, Krauss J (2020). Digital Care for Chronic Musculoskeletal Pain: 10,000 Participant Longitudinal Cohort Study. J Med Internet Res.

[50] Watson NL, Mull KE, Heffner JL, McClure JB, Bricker JB (2018). Participant Recruitment and Retention in Remote eHealth Intervention Trials: Methods and Lessons Learned From a Large Randomized Controlled Trial of Two Web-Based Smoking Interventions. J Med Internet Res.

[51] Bott N, Kumar S, Krebs C, Glenn JM, Madero EN, Juusola JL (2018). A Remote Intervention to Prevent or Delay Cognitive Impairment in Older Adults: Design, Recruitment, and Baseline Characteristics of the Virtual Cognitive Health (VC Health) Study. JMIR Res Protoc.

[52] Hamilton FL, Hornby J, Sheringham J, Linke S, Ashton C, Moore K, Stevenson F, Murray E (2018). DIAMOND (DIgital Alcohol Management ON Demand): a feasibility RCT and embedded process evaluation of a digital health intervention to reduce hazardous and harmful alcohol use recruiting in hospital emergency departments and online. Pilot Feasibility Stud.

[53] Abbate KJ, Hingle MD, Armin J, Giacobbi P, Gordon JS (2017). Recruiting Women to a Mobile Health Smoking Cessation Trial: Low- and No-Cost Strategies. JMIR Res Protoc.

[54] Druce KL, McBeth J, van der Veer SN, Selby DA, Vidgen B, Georgatzis K, Hellman B, Lakshminarayana R, Chowdhury A, Schultz DM, Sanders C, Sergeant JC, Dixon WG (2017). Recruitment and Ongoing Engagement in a UK Smartphone Study Examining the Association Between Weather and Pain: Cohort Study. JMIR Mhealth Uhealth.

[55] Gordon JS, Armin J, Giacobbi P, Cunningham JK, Johnson T, Abbate K, Howe CL, Roe DJ (2017). Development and evaluation of the See Me Smoke-Free multi-behavioral mHealth app for women smokers. Transl Behav Med.

[56] Laws RA, Litterbach EV, Denney-Wilson EA, Russell CG, Taki S, Ong K, Elliott RM, Lymer SJ, Campbell KJ (2016). A Comparison of Recruitment Methods for an mHealth Intervention Targeting Mothers: Lessons from the Growing Healthy Program. J Med Internet Res.

[57] Ashford MT, Olander EK, Rowe H, Fisher JR, Ayers S (2018). Feasibility and Acceptability of a Web-Based Treatment with Telephone Support for Postpartum Women With Anxiety: Randomized Controlled Trial. JMIR Ment Health.

[58] Fleischmann RJ, Harrer M, Zarski A, Baumeister H, Lehr D, Ebert DD (2018). Patients' experiences in a guided Internet- and App-based stress intervention for college students: A qualitative study. Internet Interv.

[59] Bidargaddi N, Musiat P, Winsall M, Vogl G, Blake V, Quinn S, Orlowski S, Antezana G, Schrader G (2017). Efficacy of a Web-Based Guided Recommendation Service for a Curated List of Readily Available Mental Health and Well-Being Mobile Apps for Young People: Randomized Controlled Trial. J Med Internet Res.

[60] Edney S, Ryan JC, Olds T, Monroe C, Fraysse F, Vandelanotte C, Plotnikoff R, Curtis R, Maher C (2019). User Engagement and Attrition in an App-Based Physical Activity Intervention: Secondary Analysis of a Randomized Controlled Trial. J Med Internet Res.

[61] Chan YY, Wang P, Rogers L, Tignor N, Zweig M, Hershman SG, Genes N, Scott ER, Krock E, Badgeley M, Edgar R, Violante S, Wright R, Powell CA, Dudley JT, Schadt EE (2017). The Asthma Mobile Health Study, a large-scale clinical observational study using ResearchKit. Nat Biotechnol.

[62] Chernick L (2021). Improving Adolescent Sexual and Reproductive Health: Can Mobile Health Interventions Affect Behavior?. Pediatrics.

[63] Short CE, Rebar A, James EL, Duncan MJ, Courneya KS, Plotnikoff RC, Crutzen R, Vandelanotte C (2017). How do different delivery schedules of tailored web-based physical activity advice for breast cancer survivors influence intervention use and efficacy?. J Cancer Surviv.

[64] Zarski A, Lehr D, Berking M, Riper H, Cuijpers P, Ebert DD (2016). Adherence to Internet-Based Mobile-Supported Stress Management: A Pooled Analysis of Individual Participant Data From Three Randomized Controlled Trials. J Med Internet Res.

[65] Poppe L, Van der Mispel C, Crombez G, De Bourdeaudhuij I, Schroé H, Verloigne M (2018). How Users Experience and Use an eHealth Intervention Based on Self-Regulation: Mixed-Methods Study. J Med Internet Res.

[66] Zlotorzynska M, Bauermeister JA, Golinkoff JM, Lin W, Sanchez TH, Hightow-Weidman L (2021). Online recruitment of youth for mHealth studies. Mhealth.

[67] Garabedian LF, Ross-Degnan D, LeCates RF, Wharam JF (2019). Uptake and use of a diabetes management program with a mobile glucometer. Prim Care Diabetes.

[68] Korinek EV, Phatak SS, Martin CA, Freigoun MT, Rivera DE, Adams MA, Klasnja P, Buman MP, Hekler EB (2018). Adaptive step goals and rewards: a longitudinal growth model of daily steps for a smartphone-based walking intervention. J Behav Med.

[69] Schneider RB, Omberg L, Macklin EA, Daeschler M, Bataille L, Anthwal S, Myers TL, Baloga E, Duquette S, Snyder P, Amodeo K, Tarolli CG, Adams JL, Callahan KF, Gottesman J, Kopil CM, Lungu C, Ascherio A, Beck JC, Biglan K, Espay AJ, Tanner C, Oakes D, Shoulson I, Novak D, Kayson E, Ray Dorsey E, Mangravite L, Schwarzschild MA, Simuni T, Parkinson Study Group AT-HOME PD Investigators (2021). Design of a virtual longitudinal observational study in Parkinson's disease (AT-HOME PD). Ann Clin Transl Neurol.

[70] Kim H, Ray CD, Veluscek AM (2017). Complementary Support from Facilitators and Peers for Promoting mHealth Engagement and Weight Loss. J Health Commun.

[71] Baca-Motes K, Edwards AM, Waalen J, Edmonds S, Mehta RR, Ariniello L, Ebner GS, Talantov D, Fastenau JM, Carter CT, Sarich TC, Felicione E, Topol EJ, Steinhubl SR (2019). Digital recruitment and enrollment in a remote nationwide trial of screening for undiagnosed atrial fibrillation: Lessons from the randomized, controlled mSToPS trial. Contemp Clin Trials Commun.

[72] Richards D, Murphy T, Viganó N, Timulak L, Doherty G, Sharry J, Hayes C (2016). Acceptability, satisfaction and perceived efficacy of "Space from Depression": an internet-delivered treatment for depression. Internet Interv.

[73] Schoenfelder E, Moreno M, Wilner M, Whitlock KB, Mendoza JA (2017). Piloting a mobile health intervention to increase physical activity for adolescents with ADHD. Prev Med Rep.

[74] Schlosser DA, Campellone TR, Truong B, Anguera JA, Vergani S, Vinogradov S, Arean P (2017). The feasibility, acceptability, and outcomes of PRIME-D: A novel mobile intervention treatment for depression. Depress Anxiety.

[75] Pratap A, Grant D, Vegesna A, Tummalacherla M, Cohan S, Deshpande C, Mangravite L, Omberg L (2020). Evaluating the Utility of Smartphone-Based Sensor Assessments in Persons With Multiple Sclerosis in the Real-World Using an App (elevateMS): Observational, Prospective Pilot Digital Health Study. JMIR Mhealth Uhealth.

[76] Williamson GR, O'Connor A, Chamberlain C, Halpin D (2018). mHealth resources for asthma and pregnancy care: Methodological issues and social media recruitment. A discussion paper. J Adv Nurs.

[77] Blake H, Suggs LS, Coman E, Aguirre L, Batt ME (2017). Active8! Technology-Based Intervention to Promote Physical Activity in Hospital Employees. Am J Health Promot.

[78] Keadle SK, Meuter L, Phelan S, Phillips SM (2021). Charity-based incentives motivate young adult cancer survivors to increase physical activity: a pilot randomized clinical trial. J Behav Med.

[79] Mitchell M, White L, Lau E, Leahey T, Adams MA, Faulkner G (2018). Evaluating the Carrot Rewards App, a Population-Level Incentive-Based Intervention Promoting Step Counts Across Two Canadian Provinces: Quasi-Experimental Study. JMIR Mhealth Uhealth.

[80] Pratap A, Renn BN, Volponi J, Mooney SD, Gazzaley A, Arean PA, Anguera JA (2018). Using Mobile Apps to Assess and Treat Depression in Hispanic and Latino Populations: Fully Remote Randomized Clinical Trial. J Med Internet Res.

[81] Edney S, Looyestyn J, Ryan J, Kernot J, Maher C (2018). Posts, pics, or polls? Which post type generates the greatest engagement in a Facebook physical activity intervention?. Transl Behav Med.

[82] Food and Drug Administration (FDA) (2000). Spectrum of Diseases/Conditions. FDA.

[83] Meyerowitz-Katz G, Ravi S, Arnolda L, Feng X, Maberly G, Astell-Burt T (2020). Rates of Attrition and Dropout in App-Based Interventions for Chronic Disease: Systematic Review and Meta-Analysis. J Med Internet Res.

[84] Lie SS, Karlsen B, Oord ER, Graue M, Oftedal B (2017). Dropout From an eHealth Intervention for Adults With Type 2 Diabetes: A Qualitative Study. J Med Internet Res.

[85] Becker S, Miron-Shatz T, Schumacher N, Krocza J, Diamantidis C, Albrecht U (2014). mHealth 2.0: Experiences, Possibilities, and Perspectives. JMIR Mhealth Uhealth.

[86] Pratt-Chapman M, Moses J, Arem H (2021). Strategies for the Identification and Prevention of Survey Fraud: Data Analysis of a Web-Based Survey. JMIR Cancer.

[87] Glazer JV, MacDonnell K, Frederick C, Ingersoll K, Ritterband LM (2021). Liar! Liar! Identifying eligibility fraud by applicants in digital health research. Internet Interv.

[88] Kolovson S, Pratap A, Duffy J, Allred R, Munson SA, Areán PA (2020). Understanding Participant Needs for Engagement and Attitudes towards Passive Sensing in Remote Digital Health Studies. Int Conf Pervasive Comput Technol Healthc.

[89] Baumel A, Muench FJ (2021). Effort-Optimized Intervention Model: Framework for Building and Analyzing Digital Interventions That Require Minimal Effort for Health-Related Gains. J Med Internet Res.

[90] Druce KL, Dixon WG, McBeth J (2019). Maximizing Engagement in Mobile Health Studies: Lessons Learned and Future Directions. Rheum Dis Clin North Am.

[91] Rawstorn JC, Gant N, Rolleston A, Whittaker R, Stewart R, Benatar J, Warren I, Meads A, Jiang Y, Maddison R (2018). End Users Want Alternative Intervention Delivery Models: Usability and Acceptability of the REMOTE-CR Exercise-Based Cardiac Telerehabilitation Program. Arch Phys Med Rehabil.

[92] Partridge S, Redfern J (2018). Strategies to Engage Adolescents in Digital Health Interventions for Obesity Prevention and Management. Healthcare.

[93] Tebb KP, Leng Trieu S, Rico R, Renteria R, Rodriguez F, Puffer M (2019). A Mobile Health Contraception Decision Support Intervention for Latina Adolescents: Implementation Evaluation for Use in School-Based Health Centers. JMIR Mhealth Uhealth.

[94] Dulli L, Ridgeway K, Packer C, Murray KR, Mumuni T, Plourde KF, Chen M, Olumide A, Ojengbede O, McCarraher DR (2020). A Social Media-Based Support Group for Youth Living With HIV in Nigeria (SMART Connections): Randomized Controlled Trial. J Med Internet Res.

[95] Verbiest MEA, Corrigan C, Dalhousie S, Firestone R, Funaki T, Goodwin D, Grey J, Henry A, Humphrey G, Jull A, Vano M, Pekepo C, Morenga LT, Whittaker R, Mhurchu CN (2019). Using codesign to develop a culturally tailored, behavior change mHealth intervention for indigenous and other priority communities: A case study in New Zealand. Transl Behav Med.

[96] McConnell MV, Shcherbina A, Pavlovic A, Homburger JR, Goldfeder RL, Waggot D, Cho MK, Rosenberger ME, Haskell WL, Myers J, Champagne MA, Mignot E, Landray M, Tarassenko L, Harrington RA, Yeung AC, Ashley EA (2017). Feasibility of Obtaining Measures of Lifestyle From a Smartphone App: The MyHeart Counts Cardiovascular Health Study. JAMA Cardiol.

[97] Huberman B, Adar E, Fine L (2005). Valuating Privacy. IEEE Secur. Privacy Mag.

[98] Heidel A, Hagist C, Schlereth C (2021). Pricing through health apps generated data-Digital dividend as a game changer: Discrete choice experiment. PLoS One.

[99] Vlaev I, King D, Darzi A, Dolan P (2019). Changing health behaviors using financial incentives: a review from behavioral economics. BMC Public Health.

[100] Promberger M, Dolan P, Marteau TM (2012). "Pay them if it works": discrete choice experiments on the acceptability of financial incentives to change health related behaviour. Soc Sci Med.

[101] Short K, Chadwick J, Cannady T, Branam D, Wharton D, Tullier M, Thompson D, Copeland K (2018). Using financial incentives to promote physical activity in American Indian adolescents: A randomized controlled trial. PLoS ONE.

[102] Voyer B (2015). ‘Nudging’ behaviours in healthcare: Insights from behavioural economics. British Journal of Healthcare Management.

[103] van Roy R, Zaman B (2017). Why Gamification Fails in Education and How to Make It Successful: Introducing Nine Gamification Heuristics Based on Self-Determination Theory. Serious Games and Edutainment Applications.

[104] Martin A, Caon M, Adorni F, Andreoni G, Ascolese A, Atkinson S, Bul K, Carrion C, Castell C, Ciociola V, Condon L, Espallargues M, Hanley J, Jesuthasan N, Lafortuna CL, Lang A, Prinelli F, Puidomenech Puig E, Tabozzi SA, McKinstry B (2020). A Mobile Phone Intervention to Improve Obesity-Related Health Behaviors of Adolescents Across Europe: Iterative Co-Design and Feasibility Study. JMIR Mhealth Uhealth.

[105] O'Connor S, Hanlon P, O'Donnell CA, Garcia S, Glanville J, Mair FS (2016). Understanding factors affecting patient and public engagement and recruitment to digital health interventions: a systematic review of qualitative studies. BMC Med Inform Decis Mak.

[106] Glasgow RE, Nelson CC, Kearney KA, Reid R, Ritzwoller DP, Strecher VJ, Couper MP, Green B, Wildenhaus K (2007). Reach, engagement, and retention in an Internet-based weight loss program in a multi-site randomized controlled trial. J Med Internet Res.

[107] Jakob R, Harperink S, Rudolf AM, Fleisch E, Haug S, Mair JL, Salamanca-Sanabria A, Kowatsch T (2022). Factors Influencing Adherence to mHealth Apps for Prevention or Management of Noncommunicable Diseases: Systematic Review. J Med Internet Res.

[108] Denney-Wilson E, Laws R, Russell CG, Ong K, Taki S, Elliot R, Azadi L, Lymer S, Taylor R, Lynch J, Crawford D, Ball K, Askew D, Litterbach EK, J Campbell K (2015). Preventing obesity in infants: the Growing healthy feasibility trial protocol. BMJ Open.

[109] Richards D, Timulak L, O'Brien E, Hayes C, Vigano N, Sharry J, Doherty G (2015). A randomized controlled trial of an internet-delivered treatment: Its potential as a low-intensity community intervention for adults with symptoms of depression. Behav Res Ther.

[110] Richards D, Timulak L, Doherty G, Sharry J, Colla A, Joyce C, Hayes C (2014). Internet-delivered treatment: its potential as a low-intensity community intervention for adults with symptoms of depression: protocol for a randomized controlled trial. BMC Psychiatry.

[111] Bricker JB, Mull KE, McClure JB, Watson NL, Heffner JL (2018). Improving quit rates of web-delivered interventions for smoking cessation: full-scale randomized trial of WebQuit.org versus Smokefree.gov. Addiction.

[112] Edney S, Plotnikoff R, Vandelanotte C, Olds T, De Bourdeaudhuij I, Ryan J, Maher C (2017). "Active Team" a social and gamified app-based physical activity intervention: randomised controlled trial study protocol. BMC Public Health.

[113] Ybarra M, Goodenow C, Rosario M, Saewyc E, Prescott T (2021). An mHealth Intervention for Pregnancy Prevention for LGB Teens: An RCT. Pediatrics.

[114] Looyestyn J, Kernot J, Boshoff K, Maher C (2018). A Web-Based, Social Networking Beginners' Running Intervention for Adults Aged 18 to 50 Years Delivered via a Facebook Group: Randomized Controlled Trial. J Med Internet Res.

[115] Heber E, Ebert DD, Lehr D, Nobis S, Berking M, Riper H (2013). Efficacy and cost-effectiveness of a web-based and mobile stress-management intervention for employees: design of a randomized controlled trial. BMC Public Health.

[116] Dalton C, Carlson S, Butler M, Cassano D, Clarke S, Fejsa J, Durrheim D (2017). Insights From Flutracking: Thirteen Tips to Growing a Web-Based Participatory Surveillance System. JMIR Public Health Surveill.

[117] De Brouwer W, Patel CJ, Manrai AK, Rodriguez-Chavez IR, Shah NR (2021). Empowering clinical research in a decentralized world. NPJ Digit Med.

[118] Apostolaros M, Babaian D, Corneli A, Forrest A, Hamre G, Hewett J, Podolsky L, Popat V, Randall P (2020). Legal, Regulatory, and Practical Issues to Consider When Adopting Decentralized Clinical Trials: Recommendations From the Clinical Trials Transformation Initiative. Ther Innov Regul Sci.

[119] Field N, Cohen T, Struelens MJ, Palm D, Cookson B, Glynn JR, Gallo V, Ramsay M, Sonnenberg P, Maccannell D, Charlett A, Egger M, Green J, Vineis P, Abubakar I (2014). Strengthening the Reporting of Molecular Epidemiology for Infectious Diseases (STROME-ID): an extension of the STROBE statement. Lancet Infect Dis.

[120] Agarwal S, LeFevre AE, Lee J, L'Engle K, Mehl G, Sinha C, Labrique A, WHO mHealth Technical Evidence Review Group (2016). Guidelines for reporting of health interventions using mobile phones: mobile health (mHealth) evidence reporting and assessment (mERA) checklist. BMJ.

