# Supplementary material for: Enrollment and Retention of Participants in Remote Digital Health Studies: Scoping Review and Framework Proposal
Source: J Med Internet Res. 2022 Sep 9;24(9):e39910. doi: 10.2196/39910 (PMC9508669; doi:10.2196/39910)
Supplement: Multimedia Appendix 1 [file jmir_v24i9e39910_app1.docx]

**Multimedia Appendix 1**: Methods and additional descriptive results

Additional information for “Conceptual framework and hypotheses” subsection in the **Introduction**

Our framework is guided by recent evidence on participatory patterns in digital health research. Specifically, we built on the possible factors that influence study enrollment and retention proposed by Eysenbach [16]. We then adapted these factors based on recent evidence from our studies and large-scale remote digital health studies on study enrollment and retention (**eTable 1**). We refer specifically to the widely reported Apple Heart Study [5] and a cross-study evaluation of large-scale remote digital health studies [19]. From these studies, we detected a pattern of high participant enrollment through mobile-forward recruitment strategies but a greater risk of low study task completion after enrollment in remote digital health studies.

We further identified possible benefits on remote digital health study outcomes when varying participant profiles are considered in study planning. Specifically, the propensity for participants to complete study tasks can be affected by varying participant motivations and task complexities [16,19]. We further build on this concept with the 90-9-1 principle for participatory patterns within Internet communities [28]. The principle estimates that 90% of social network users will not contribute, 9% will contribute occasionally, and 1% will contribute to most of new content. The 90-9-1 principle has been used alongside the diffusion of innovation theory (DIT) to explain participation inequality in healthcare and digital health research [22,23]. Applied to remote digital health studies, a majority of enrolled participants may contribute minimally, a smaller fraction may provide some contributions, and a small fraction may provide high contributions to a study.

The development of the framework was, lastly, motivated by our practical experiences in planning and conducting two longitudinal remote digital health studies. In these studies, we observed a correlation between study task completion and the required tasks and incentives required to motivate participants [2,20]. Based on these guiding principles and experiences with remote digital health research, we identified three overarching themes that encompass most factors that should be considered by investigators when planning remote digital health studies.

**eTable 1.** Framework criteria and factors affecting study outcomes based on literature

| **Framework criteria** | **Associated loss to follow-up factors** |
| --- | --- |
| Motivation profile of participants and offered incentives or nudges | - Expectation management [16,19-21] - Usability and interface ease-of-use [5,16,21] - Push factors [16] - (Lack of) personal contact [16,19,21] - Encouragement from change agents [16,19,21] - Compensation for trial completion [16,19] - Competing interventions [16] - External events [16,21] - Community building [16,19,20,21] - Altruistic motives for joining study [19-21] - Trust in scientific team and research [19-21] - In-app communication [19] - Return of information to patients [19-21] - Adapted participant targeting [19,21] *(90-9-1 principle and diffusion of innovation approach)* |
| Complexity of tasks required from participants | - Ease of enrollment [5,16,19-21] - Ease of dropout [5,16,19-21] - Workload and time required [5,16,19-21] - Experience of the user [16,20,21] - Digital divide [5,19-21] - Digital literacy and affinity [5,19-21] - Appeal of passive monitoring [5] |
| Scientific requirements of the study | - Revision of target sample and size for statistical power [19] - Enrollment study design for representative samples [19-21] - Varying trial withdrawal designs [19] |

Additional information for “Study outcome definitions” section in the **Introduction**

Based on the proposed criteria by Eysenbach and recent research on attrition patterns in digital health studies [14,24,25] we identified recruitment and retention as two key phases of digital health research that influence participant attrition or dropout. From our research, we also identified difficulties with technology adoption as a main contributor to high participant dropout in digital health studies [26]. While virtual contact with participants at enrollment is mentioned as a potential motivator for participant dropout [16], we observed a noticeable gap in literature on steps that researchers may take to provide (technical) assistance and support to participants at enrollment. We define this process as onboarding. As such, we refer to three phases of digital health studies that impact participant attrition: 1) recruitment, 2) onboarding and 3) retention.

Within these three phases, we identified two outcomes that are widely reported in digital health studies: 1) study enrollment and 2) study completion, based predominantly on the CONSORT EHEALTH reporting criteria [27]. More specifically, study enrollment is a measure of the number of participants who are successfully recruited in the study, provide informed consent and complete the required baseline assessments. Study completion is a measure of participants with full follow-through of the study procedure and required tasks based on completion criteria defined a priori in the study protocol.
