# Supplementary material for: Enrollment and Retention of Participants in Remote Digital Health Studies: Scoping Review and Framework Proposal
Source: J Med Internet Res. 2022 Sep 9;24(9):e39910. doi: 10.2196/39910 (PMC9508669; doi:10.2196/39910)
Supplement: Multimedia Appendix 3 [file jmir_v24i9e39910_app3.docx]

**Multimedia Appendix 3:** Search Strategy

*PubMed (first two search categories in title and abstract, last search category in full text)*

(“digital health stud*”[Title/Abstract] OR “digital health intervention*”[Title/Abstract] OR “remote stud*”[Title/Abstract] OR “remote intervention”[Title/Abstract] OR “eHealth stud*”[Title/Abstract] OR “eHealth intervention”[Title/Abstract] OR “digital trial”[Title/Abstract] OR “mHealth stud*”[Title/Abstract] OR “mHealth intervention*”[Title/Abstract] OR “mobile health stud*”[Title/Abstract] OR “mobile health intervention*”[Title/Abstract] OR “site-less stud*”[Title/Abstract] OR “eCohort*”[Title/Abstract] OR “digital cohort*”[Title/Abstract]) AND (“retention”[Title/Abstract] OR “retain*”[Title/Abstract] OR “online recruit*”[Title/Abstract] OR “digital recruit*”[Title/Abstract] OR “onboard*”[Title/Abstract] OR “enrol*”[Title/Abstract] OR “attrition”[Title/Abstract] OR “nonparticipat*”[Title/Abstract] OR “non-participat*”[Title/Abstract] OR “abandon*”[Title/Abstract] OR “drop-out”[Title/Abstract] OR “dropout”[Title/Abstract] OR “participat*”[Title/Abstract] OR “engage*”[Title/Abstract]) AND (“reward*” OR “incentive*” OR “motivation*” OR “complex*” OR “complicat*” OR “challeng*” OR “barrier*” OR “facilitat*” OR “difficult*” OR “effort*” OR “sample size*” OR “sample require*” OR “sampling strateg*” OR “sample characteristic*” OR “participant characteristic*” OR “task completion”)

*CINAHL (first two search categories in title and abstract, last search category in full text)*

((“digital health” W2 “stud*”) OR (“digital health” W2 “intervention*”) OR (“remote” W2 “stud*”) OR (“remote” W2 “intervention”) OR “eHealth stud*” OR “eHealth intervention” OR (“digital” W2 “trial”) OR “mHealth stud*” OR “mHealth intervention*” OR “mobile health stud*” OR “mobile health intervention*” OR (“site-less” W2 “stud*”) OR “eCohort*” OR “digital cohort*”) AND (“retention” OR “retain*” OR “online recruit*” OR “digital recruit*” OR “onboard*” OR “enrol*” OR “attrition” OR “nonparticipat*” OR “non-participat*” OR “abandon*” OR “drop-out” OR “dropout” OR “participat*” OR “engage*”) AND (“reward*” OR “incentive*” OR “motivation*” OR “complex*” OR “complicat*” OR “challeng*” OR “barrier*” OR “facilitat*” OR “difficult*” OR “effort*” OR “sample size*” OR “sample require*” OR “sampling strateg*” OR “sample characteristic*” OR “participant characteristic*” OR “task completion”)

*Web of Science* *(only topic search i.e., title, abstract and keywords)*

((“digital health research” OR (“digital health” NEAR/2 “stud*”) OR (“digital health” NEAR/2 “intervention*”) OR (“remote” NEAR/2 “stud*”) OR (“remote” NEAR/2 “intervention”) OR “eHealth stud*” OR “eHealth intervention” OR (“digital” NEAR/2 “trial”) OR “mHealth stud*” OR “mHealth intervention*” OR “mobile health stud*” OR “mobile health intervention*” OR (“site-less” NEAR/2 “stud*”) OR “eCohort*” OR “digital cohort*”) AND (“retention” OR “retain*” OR “online recruit*” OR “digital recruit*” OR “onboard*” OR “enrol*” OR “attrition” OR “nonparticipat*” OR “non-participat*” OR “abandon*” OR “drop-out” OR “dropout” OR “participat*” OR “engage*”) AND (“reward*” OR “incentive*” OR “motivation*” OR “complex*” OR “complicat*” OR “challeng*” OR “barrier*” OR “facilitat*” OR “difficult*” OR “effort*” OR “sample size*” OR “sample require*” OR “sampling strateg*” OR “sample characteristic*” OR “participant characteristic*” OR “task completion”))
