# Supplementary material for: Enrollment and Retention of Participants in Remote Digital Health Studies: Scoping Review and Framework Proposal
Source: J Med Internet Res. 2022 Sep 9;24(9):e39910. doi: 10.2196/39910 (PMC9508669; doi:10.2196/39910)
Supplement: Multimedia Appendix 4 [file jmir_v24i9e39910_app4.docx]

**Multimedia Appendix 4.** General characteristics of included studies

| **Reference** | **Study Design** | **Study Type** | **Therapeutic Area^a^** | **Digital Technology** | **Digital Technology Endpoint** | **Patient Reported Outcomes** | **Study Conductor** | **Age^b^** | **Gender^c^** | **Education^d^** | **Income^e^** | **Employment Status^f^** |
| --- | --- | --- | --- | --- | --- | --- | --- | --- | --- | --- | --- | --- |
| **Study duration <= 12 weeks** | | | | | | | | | | | | |
| Keadle et al., 2021 [78] | RCT | Intervention | Oncology | Wearable (Fitbit) | Step counts | Yes (PROMIS, PSQI) | Academy | 37 | 86% | 33% | NA | 14% |
| Pratap et al., 2020 [75] | Non-experimental study | Observational | Neurology | Smartphone (app) | MS health status, Quality of life | Yes (PDDS, Neuro-QoL, functional tests) | Academy / Industry | 45 | 41% | 4% | NA | 2% |
| Bailey et al., 2020 [49] | Non-experimental study | Observational | Analgesia, Psychiatry | Smartphone / Tablet (app), Wearable (accelerator and gyrometer) | Pain assessment, Mental health | Yes (VAS, PHQ-9, GAD-7, WPAI, KOOS, MvK) | Academy / Industry | 44 | 50% | NA | NA | NA |
| Edney et al., 2018 [81] | RCT | Intervention | Prevention | Computer (social media) | Physical activity (self-report) | Yes (AAS, Self-Efficacy Barriers to Exercise Measure, Exercise Attitude Questionnaire, social support and exercise survey) | Academy | 35 | 80% | 27% | NA | NA |
| Pratap et al., 2018 [80] | RCT | Intervention | Psychiatry | Smartphone (app) | Mental health management | Yes (PHQ-9, SDS, PHQ-2) | Academy / Industry | 35 | 77% | 37% | 29.6% | NA |
| Poppe et al., 2018 [65] | RCT | Intervention | Prevention | Computer (web app) | Physical activity | Yes (IPAQ, Intervention) | Academy | 47 | 50% | 50% | NA | NA |
| Ashford et al., 2018 [57] | RCT | Intervention | Psychiatry | Computer (web app) | Anxiety management | Yes (DASS-21, GAD-7) | Academy | 32 | 100% | 25% | 7.9% | 72% |
| Mitchell et al., 2018 [79] | Quasi-experimental | Intervention | Prevention | Smartphone (app) | Step counts | NA | Academy / Industry | 34 | 66% | NA | 29700 CAD / year **^g^** | NA |
| Crouthamel et al., 2018 [7] | Quasi-experimental | Observational | Rheumatology | Smartphone (app) | Disease status tracking | Yes (Rheumatoid Arthritis Severity Scale, EuroQoL, Health Assessment Questionnaire-Disability Index) | Industry | 48 | 81% | 23% | NA | NA |
| Abbate et al., 2017 [53] | Quasi-experimental | Intervention | Addiction | Smartphone (app) | Health outcomes based on smoking | Yes (Intervention questionnaires, Fagerstrom, BACL, CLCQ, AESMBSRQ, LTEQ) | Academy | 39 | 100% | 33% | NA | NA |
| Gordon et al., 2017 [55] | Quasi-experimental | Intervention | Addiction | Smartphone (app) | Health outcomes based on smoking | Yes (Intervention questionnaires, Fagerstrom, BACL, CLCQ, AESMBSRQ, LTEQ) | Academy | 39 | 100% | 33% | NA | NA |
| Fleischmann et al., 2017 [58] | Non-experimental study | Intervention | Prevention | Computer (web app), Smartphone (app) | Stress management | Yes (PSS-4) | Academy | 24 | 74% | 100% | NA | NA |
| Bidargaddi et al., 2017 [59] | RCT | Intervention | Prevention | Computer (web app), Smartphone (app) | Well-being and mental health | Yes (MHC-SF, EMA) | Academy / Industry | 23 | 77% | NA | NA | NA |
| Schoenfelder et al., 2017 [73] | Non-experimental study | Intervention | Psychiatry | Wearable (Fitbit), Computer (social media) | ADHD symptoms, Step counts | Yes (PANAS-C) | Academy | 16 | 54% | 100% | NA | NA |
| Short et al., 2017 [63] | RCT | Intervention | Oncology | Computer (web app) | Physical activity | Yes (GLTEQ) | Academy | 55 | 100% | 52% | 26% | 53% |
| Schlosser et al., 2017 [74] | Non-experimental study | Intervention | Psychiatry | Smartphone (all) | Mental health | Yes (PHQ-9, GAD-7, SDS) | Academy | 31 | 78% | 44% | 50% | 75% |
| Richards et al., 2016 [72] | RCT | Intervention | Prevention | Computer (web app) | Depression management | Yes (BDI-II, DSM-IV; Baseline: GAD-7, Work and Social Adjustment Scale) | Academy / Industry | 38 | 75% | 44% | NA | 30% |
| Blake et al., 2017 [77] | RCT | Intervention | Psychiatry | Smartphone (SMS), Computer (e-mail) | Physical activity | Yes (GPAQ, TPB, HRQoL, SF-12; Baseline: PAR-Q) | Academy | 39 | 86% | NA | NA | 0% |
| Zarski et al., 2016 [64] | RCT | Intervention | Prevention | Computer (web app) | Stress management, mental health | Yes (PSS-10, CES-D, HADS, MBI-GS-D, ERSQ-27, ERSQ-ES, ATSPPHS-SF, UWES, REQ, ISI, PSWQ SF-12, EQ-5D, ERI-SF, PATHEV, CSQ-8) | Academy | 42 | 78% | 42% | 26.6% | 1% |
| **Study duration > 12 weeks** | | | | | | | | | | | | |
| Hernandez-Ramos et al., 2021 [43] | RCT | Intervention | Endocrinology, Psychiatry | Smartphone (app) | Step counts, mental health | Yes (PHQ-8) | Academy | 51 | 36% | 64% | NA | 18% |
| Schneider et al., 2021 [69] | Non-experimental study | Observational | Neurology | Smartphone (app), Computer (video visits) | Motor tasks | Yes (MDS-UPDRS parts IB and II, PGI, Self-reported falls, PDQ-8, NMS-QUEST, PDAQ-15, EQ-5D, PD-PROP, PASE, GDS-15) | Academy | NA | NA | NA | NA | NA |
| Chernick, 2021 [62] | RCT | Intervention | Prevention | Smartphone (SMS) | Interactive content for pregnancy prevention | Yes (Intervention questionnaires) | Academy | 16 | 100% | 100% | NA | NA |
| Damschroder et al., 2020 [48] | RCT | Intervention | Prevention | Smartphone (app), Wearable (Fitbit), Digital scale | Active minutes, step counts (secondary) | Yes (PAM) | Academy | 40 | 90% | 23% | 49% | 43% |
| Baca-Motes et al., 2019 [71] | RCT | Intervention* | Cardiology | Wearable (ZIO XT Patch) | ECG monitoring | NA | Academy / Industry | 74 | 41% | NA | NA | NA |
| Garabedian et al., 2019 [67] | Non-experimental study | Observational | Endocrinology | Mobile digital glucometer | Glucose data | NA | Academy | 54 | 44% | NA | NA | 0% |
| Edney et al., 2019 [60] | RCT | Intervention | Prevention | Smartphone (app), Wearable (accelerometer) | Step counts | Yes (AAS) | Academy | 42 | 74% | 47% | NA | NA |
| Watson et al., 2018 [50] | RCT | Intervention | Addiction, Psychiatry | Computer (web app) | Smoking cessation | Yes (Intervention (PPA); Baseline: CES-D, GAD-8, ANSQ, PCL-6, SPIN, FTND) | Academy / Industry | 46 | 79% | 28% | 28.0% | NA |
| Bott et al., 2018 [51] | Quasi-experimental | Intervention | Neurology, Psychiatry | Computer (web app) | Cognitive impairment, Mental health | Yes (SCD-9, RBANS; Baseline: PHQ-9, GAD-7) | Academy / Industry | 64 | 61% | 42% | NA | NA |
| Hamilton et al., 2018 [52] | RCT | Intervention | Addiction, Psychiatry | Computer (web app) | Alcohol cessation and management | Yes (TOT-AL, AUDIT, CORE-10, SCQ-8; Baseline: LDQ) | Academy | NA | NA | NA | NA | NA |
| Korinek et al., 2018 [68] | Quasi-experimental | Intervention | Prevention | Smartphone (app), Wearable (Fitbit) | Step counts | Yes (Intervention questionnaires; Baseline: PAR-Q) | Academy | 47 | 90% | NA | NA | NA |
| Druce et al., 2017 [54] | Non-experimental study | Observational | Analgesia | Smartphone (app) | Pain assessment | Yes (Intervention questionnaires) | Academy | 49 | 81% | NA | NA | NA |
| Kim et al., 2017 [70] | Non-experimental study | Intervention | Prevention | Smartphone (app) | Weight loss | Yes (Medical Outcomes Study social support scale) | Academy | 35 | 73% | NA | NA | NA |
| Chan et al., 2017 [61] | Non-experimental study | Observational | Pulmonary | Smartphone (app) | Asthma prevalence | Yes (Intervention questionnaires, EQ-5D, GINA) | Academy / Industry | 18-34**^h^** (54%), 35-64 (41%), 65+ (5%) | 39% | 48% | 6% | NA |
| Bot et al., 2016 [6] | Non-experimental study | Observational | Neurology | Smartphone (all) | Parkinson’s disease symptom tracking | Yes (PD assessment, PDQ-8, MDS-UPDRS) | Academy / Industry | 36 | 22% | 43% | NA | NA |
| **Study duration unspecified** | | | | | | | | | | | | |
| Zlotorzynska et al., 2021 [66] | RCT | Intervention | Infectious disease | Computer (web app), Smartphone (app) | HIV transmission reduction | Yes (Intervention questionnaires) | Academy | 15-24**^h^** | 0% | NA | NA | NA |
| Williamson et al., 2018 [76] | Non-experimental study | Observational | Pulmonary | Computer (web app) | Asthma prevalence | Yes (Intervention questionnaires) | Academy | NA | 100% | NA | NA | NA |
| Laws et al., 2016 [56] | Quasi-experimental | Intervention | Prevention | Computer (web app), Smartphone (app) | Improvement of healthy infant feeding practices | Yes (FFQ, IFQ) | Academy | 30 | 100% | 48% | 16% | 89% |

**^a^** Therapeutic area: classifications based on *Spectrum of Diseases by Therapeutic Area* from the Food and Drug Administration (FDA) [82]; studies that target healthy participants were labeled under an additional “Prevention” category

**^b^** Age: mean age of participants

**^c^** Gender: % female

**^d^** Education: % of participants who achieved an education below a university degree

**^e^** Income: % of participants in lowest reported income bracket in study

**^f^** Employment Status: % of participants who reported being “Unemployed” or “Other”

**^g^** Median reported yearly income

**^h^** Age ranges of participants in studies (mean age not available)

*Assessment of Baca-Motes et al., 2019 [71] in this study focused on intervention arm only
