# Supplementary material for: Enrollment and Retention of Participants in Remote Digital Health Studies: Scoping Review and Framework Proposal
Source: J Med Internet Res. 2022 Sep 9;24(9):e39910. doi: 10.2196/39910 (PMC9508669; doi:10.2196/39910)
Supplement: Multimedia Appendix 5 [file jmir_v24i9e39910_app5.docx]

**Multimedia Appendix 5.** Requirements and outcomes of included studies

| **Reference** | **Motivation Profile** | **Research Goal** | **Study Duration** | **Monthly Steps** | **Total Steps** | **Target Sample Size** | **Enrolled Participants** | **Retained Participants** | **Study Completion** |
| --- | --- | --- | --- | --- | --- | --- | --- | --- | --- |
| **Study duration =< 12 weeks** | | | | | | | | | |
| Keadle et al., 2021 [78] | Extrinsic | Confirmatory | 12 weeks | 64 | 193 | 40 | 51 | 43 | 84% |
| Pratap et al., 2020 [75] | Intrinsic | Exploratory | 12 weeks | 64 | 192 | NA | 629 | 269 | 43% |
| Bailey et al., 2020 [49] | Extrinsic | Exploratory | 12 weeks | 28 | 84 | 10000 | 10264 | 7497 | 73% |
| Edney et al., 2018 [81] | Extrinsic | Confirmatory | 8 weeks | 16 | 34 | NA | 89 | 69 | 78% |
| Pratap et al., 2018 [80] | Extrinsic | Exploratory | 12 weeks | 64 | 140 | NA | 1040 | 348 | 33% |
| Poppe et al., 2018 [65] | Extrinsic | Theory-Driven | 5 weeks | 4 | 6 | 20 | 20 | NA | NA |
| Ashford et al., 2018 [57] | Extrinsic | Exploratory | 12 weeks | 24 | 74 | 72 | 89 | 29 | 33% |
| Mitchell et al., 2018 [79] | Extrinsic | Theory-Driven | 12 weeks | 30 | 120 | NA | 35014 | 19964 | 62% |
| Crouthamel et al., 2018 [7] | Intrinsic | Exploratory | 12 weeks | 12 | 41 | NA | 399 | 45 | 11% |
| Abbate et al., 2017 [53] | Extrinsic | Exploratory | 12 weeks | 30 | 93 | 50 | 151 | 73 | 48% |
| Gordon et al., 2017 [55] | Extrinsic | Exploratory | 12 weeks | 30 | 93 | 50 | 151 | 73 | 48% |
| Fleischmann et al., 2017 [58] | Intrinsic | Theory-Driven | 12 weeks | 4 | 10 | NA | 150 | NA | NA |
| Bidargaddi et al., 2017 [59] | Intrinsic | Confirmatory | 4 weeks | 12 | 13 | NA | 387 | 194 | 50% |
| Schoenfelder et al., 2017 [73] | Extrinsic | Exploratory | 4 weeks | 16 | 16 | NA | 11 | 11 | 84% |
| Short et al., 2017 [63] | Extrinsic | Exploratory | 12 weeks | 6 | 20 | NA | 492 | 156 | 32% |
| Schlosser et al., 2017 [74] | Extrinsic | Hypothesis-Driven | 12 weeks | 4 | 35 | NA | 36 | 30 | 84% |
| Richards et al., 2016 [72] | Extrinsic | Exploratory | 8 weeks | 4 | 10 | 120 | 281 | 121 | 43% |
| Blake et al., 2017 [77] | Intrinsic | Confirmatory | 12 weeks | NA | 5 | 98 | 296 | 111 | 38% |
| Zarski et al., 2016 [64] | Extrinsic | Confirmatory | 7 weeks | 3 | 10 | 264 | 395 | 239 | 61% |
| **Study duration > 12 weeks** | | | | | | | | | |
| Hernandez-Ramos et al., 2021 [43] | Extrinsic | Theory-Driven | 26 weeks | NA | NA | 276 | 11 | NA | NA |
| Schneider et al., 2021 [69] | Intrinsic | Exploratory | 104 weeks | NA | 37 | 225 | 226 | 161 | NA |
| Chernick, 2021 [62] | Extrinsic | Confirmatory | 20 weeks | NA | 5 | NA | 948 | 799 | 84% |
| Damschroder et al., 2020 [48] | Extrinsic | Confirmatory | 52 weeks | 8 | 101 | 350 | 357 | NA | 36% |
| Baca-Motes et al., 2019 [71] | Intrinsic | Hypothesis-Driven | 52 weeks | 3 | 6 | 2000 | 2659 | 1738 | 65% |
| Garabedian et al., 2019 [67] | Intrinsic | Exploratory | 52 weeks | 30 | 365 | NA | 556 | 253 | 46% |
| Edney et al., 2019 [60] | Extrinsic | Confirmatory | 14 weeks | 30 | 106 | NA | 301 | 193 | 64% |
| Watson et al., 2018 [50] | Extrinsic | Exploratory | 52 weeks | NA | 4 | NA | 2637 | 2309 | 88% |
| Bott et al., 2018 [51] | Extrinsic | Hypothesis-Driven | 52 weeks | 28 | 342 | NA | 82 | NA | NA |
| Hamilton et al., 2018 [52] | Intrinsic | Confirmatory | 14 weeks | 38 | 97 | 20 | 19 | 5 | 16% |
| Korinek et al., 2018 [68] | Extrinsic | Hypothesis-Driven | 14 weeks | 58 | 93 | NA | 20 | NA | NA |
| Druce et al., 2017 [54] | Intrinsic | Exploratory | 26 weeks | 30 | 180 | 1000 | 6370 | NA | 100% |
| Kim et al., 2017 [70] | Extrinsic | Hypothesis-Driven | 26 weeks | NA | 1 | NA | 2877 | NA | NA |
| Chan et al., 2017 [61] | Intrinsic | Exploratory | 26 weeks | 34 | 174 | NA | 7593 | 2317 | 31% |
| Bot et al., 2016 [6] | Intrinsic | Exploratory | 26 weeks | 36 | 558 | NA | 6805 | 898 | 13% |
| **Study duration unspecified** | | | | | | | | | |
| Zlotorzynska et al., 2021 [66] | Intrinsic | Exploratory | NA | NA | NA | 745 | 102 | NA | NA |
| Williamson et al., 2018 [76] | Intrinsic | Exploratory | NA | NA | 3 | NA | 7 | NA | NA |
| Laws et al., 2016 [56] | Intrinsic | Exploratory | NA | NA | NA | 200 | 300 | NA | NA |
