# Supplementary material for: Enrollment and Retention of Participants in Remote Digital Health Studies: Scoping Review and Framework Proposal
Source: J Med Internet Res. 2022 Sep 9;24(9):e39910. doi: 10.2196/39910 (PMC9508669; doi:10.2196/39910)
Supplement: Multimedia Appendix 6 [file jmir_v24i9e39910_app6.docx]

**Multimedia Appendix 6**. Incentives or nudges and task complexity of included studies

| **Reference** | **Number of Steps - Recruitment** | **Number of Steps - Onboarding** | **Number of Monthly Steps - Retention** | **Incentives or Nudges - Recruitment** | **Incentives or Nudges - Onboarding** | **Incentives or Nudges - Retention** |
| --- | --- | --- | --- | --- | --- | --- |
| **Study duration =< 12 weeks** | | | | | | |
| Keadle et al., 2021 [78] | 6 steps | 1 step | 64 | NA | NA | Monetary, Personal Coaching or Assistance |
| Pratap et al., 2020 [75] | 4 steps | NA | 64 | Referral source | NA | Referral source |
| Bailey et al., 2020 [49] | 4 steps | 1 step | 28 | NA | NA | Personal Coaching or Assistance, Community |
| Edney et al., 2018 [81] | 4 steps | 1 step | 16 | NA | NA | Personal Coaching or Assistance, Community, Monetary |
| Pratap et al., 2018 [80] | 6 steps | 5 steps | 64 | Monetary | NA | Monetary, Reminders |
| Poppe et al., 2018 [65] | 3 steps | 1 step | 4 | NA | NA | Personal Coaching or Assistance, Goal setting, Reminders, Community |
| Ashford et al., 2018 [57] | 4 steps | NA | 24 | NA | NA | Monetary, Personal Coaching or Assistance, Reminders |
| Mitchell et al., 2018 [79] | 3 steps | NA | 30 | Monetary | NA | Gamification, Monetary |
| Crouthamel et al., 2018 [7] | 4 steps | 1 step | 12 | NA | NA | Personalized feedback |
| Abbate et al., 2017 [53] | 5 steps | 1 step | 30 | Targeted ads, Cited source | NA | Monetary, Reminders |
| Gordon et al., 2017 [55] | 6 steps | 1 step | 30 | Monetary | Personalized assistance | Monetary, Reminders, Community, Reminders |
| Fleischmann et al., 2017 [58] | 4 steps | NA | 4 | NA | NA | Community, Personal Coaching or Assistance, Reminders |
| Bidargaddi et al., 2017 [59] | 5 steps | 1 step | 12 | NA | NA | NA |
| Schoenfelder et al., 2017 [73] | 7 steps | 1 step | 16 | Referral source | NA | Community, Reminders, Monetary, Gamification |
| Short et al., 2017 [63] | 4 steps | 1 step | 6 | NA | NA | Goal setting, Reminders |
| Schlosser et al., 2017 [74] | 5 steps | 1 step | 4 | Monetary | NA | Monetary, Community, Personal Coaching or Assistance |
| Richards et al., 2016 [72] | 4 steps | 1 step | 4 | Referral source | NA | Personalized feedback, Reminders |
| Blake et al., 2017 [77] | 4 steps | 1 step | NA | NA | NA | Personal Coaching or Assistance |
| Zarski et al., 2016 [64] | 4 steps | 1 step | 3 | NA | NA | Personal Coaching or Assistance, Personal Coaching or Assistance, Reminders |
| **Study duration > 12 weeks** | | | | | | |
| Hernandez-Ramos et al., 2021 [43] | 4 steps | 2 steps | NA | Referral source, Monetary | Personalized assistance, Community | Personal Coaching or Assistance, Monetary |
| Schneider et al., 2021 [69] | 6 steps | 3 steps | NA | NA | Personalized assistance | NA |
| Chernick, 2021 [62] | 5 steps | NA | NA | NA | NA | Community, Gamification, Monetary |
| Damschroder et al., 2020 [48] | 6 steps | 3 steps | 8 | Personalized invitation | Personalized assistance | Monetary, Personal Coaching or Assistance, Goal Setting, Reminders |
| Baca-Motes et al., 2019 [71] | 4 steps | 2 steps | 3 | Reminders | Personalized assistance, Video instructions | Personal Assistance |
| Garabedian et al., 2019 [67] | 3 steps | 1 step | 30 | NA | NA | NA |
| Edney et al., 2019 [60] | 6 steps | 1 step | 30 | Community | NA | Gamification, Reminders, Community |
| Watson et al., 2018 [50] | 5 steps | NA | NA | Monetary, Vested (personal) interest, Personal contact | NA | Monetary, Multi-modal strategy, Reminders |
| Bott et al., 2018 [51] | 6 steps | 1 step | 28 | NA | Personalized assistance | Personal Coaching or Assistance, Community |
| Hamilton et al., 2018 [52] | 6 steps | 1 step | 38 | NA | NA | Monetary, Reminders |
| Korinek et al., 2018 [68] | 3 steps | NA | 58 | NA | NA | Monetary, Goal setting |
| Druce et al., 2017 [54] | 5 steps | NA | 30 | NA | NA | NA |
| Kim et al., 2017 [70] | 3 steps | NA | NA | Monetary | NA | Community |
| Chan et al., 2017 [61] | 8 steps | 1 step | 34 | NA | NA | Reminders |
| Bot et al., 2016 [6] | 6 steps | 1 step | 36 | NA | NA | Personalized feedback |
| **Study duration unspecified** | | | | | | |
| Zlotorzynska et al., 2021 [66] | 5 steps | NA | NA | Monetary, Vested (personal) interest | NA | NA |
| Williamson et al., 2018 [76] | 1 step | 1 step | NA | NA | NA | NA |
| Laws et al., 2016 [56] | 5 steps | NA | NA | Monetary | NA | Monetary |
