# Supplementary material for: Enrollment and Retention of Participants in Remote Digital Health Studies: Scoping Review and Framework Proposal
Source: J Med Internet Res. 2022 Sep 9;24(9):e39910. doi: 10.2196/39910 (PMC9508669; doi:10.2196/39910)
Supplement: Multimedia Appendix 7 [file jmir_v24i9e39910_app7.docx]

**Multimedia Appendix 7**. Associations between framework criteria and outcomes

|  | **Study Enrollment**  **(N=37)** | | | **Study Completion**  **(N=27)** |
| --- | --- | --- | --- | --- |
| **Motivation Profile and Incentives** | | | | |
| **Motivation profiles** | | p = 0.35^a^ | p = 0.06 | |
| Extrinsic (median, IQR) | | 216 (IQR: 59-834) | 62% (IQR: 43-83%) | |
| Intrinsic (median, IQR) | | 387 (IQR: 188-1644) | 41% (IQR: 20%-49%) | |
| **Offered incentives: Recruitment**^b^ | | p = 0.69 | p = 0.66 | |
| Monetary (median, IQR) | | 300 (IQR: 102-2637) | 62% (IQR: 48-83%) | |
| Referral Source (median, IQR) | | 146 (IQR: 11-368) | 43% (IQR: 43-64%) | |
| Vested interest (median, IQR) | | 2637 (IQR: 1370-2648) | 77% (IQR: 71-82%) | |
| **Offered incentives: Retention**^b^ | | p = 0.67 | p = 0.07 | |
| Monetary (median, IQR) | | 120 (IQR: 32-505) | 62% (IQR: 36-84%) | |
| Reminders (median, IQR) | | 216 (IQR: 135-419) | 46% (IQR: 33-58%) | |
| Personal contact (median, IQR) | | 120 (IQR: 59-389) | 63% (IQR: 44-77%) | |
| **Task Complexity and Study Design** | | | | |
| **Study Duration** | | p = 0.09^c^, r=0.37^d^ | p = 0.09 (r=-0.38) | |
| **Task Complexity: Study Tasks** | |  |  | |
| Total Steps (Recruitment) | | p = 0.47, r=-0.16 | p = 0.81 (r=-0.06) | |
| Total Steps (Onboarding) | | p = 0.18, r=0.30 | p = 0.65 (r=-0.11) | |
| Monthly Steps (Retention) | | p = 0.80, r=0.06 | p = 0.36 (r=-0.21) | |
| Total Steps (Retention) | | p = 0.40, r=0.19 | p = 0.16 (r=-0.32) | |
| **Scientific Requirements** | | | | |
| **Study Design** | | p = 0.03 | p = 0.23 | |
| Intervention (median, IQR) | | 216 (IQR: 74-419) | 56% (IQR: 38-79%) | |
| Observational (median, IQR) | | 629 (IQR: 399-6805) | 43% (IQR: 22-60%) | |
| **Target Sample Size** | | p < 0.01, r=0.69 | p = 0.17, r=0.42 | |

^a^ **p-value** with threshold for statistical significance at p < 0.05 (2-tailed testing) from Kruskal Wallis test.

^b^ Top three recruitment and retention incentives are reported; more information available in **eTable 4**.

^c^ **p-value** with threshold for statistical significance at p < 0.05 (2-tailed testing) from Spearman’s correlation test.

^d^ **Spearman’s rho correlation coefficient** from Spearman’s correlation test.
